# Drivers of sex differences in *Mycobacterium tuberculosis* immunoreactivity among adolescents and adults in Blantyre, Malawi

**DOI:** 10.1186/s44263-026-00302-w

**Published:** 2026-07-23

**Authors:** Mphatso D Phiri, Hannah M Rickman, Hannah Mbale, Helena RA Feasey, Marriott Nliwasa, Alvaro Schwalb, Tisungane E Mwenyenkulu, Kuzani N Mbendera, Henry C Mwandumba, Elizabeth L Corbett, S Bertel Squire, Marc YR Henrion, Katherine C Horton, Peter MacPherson

**Affiliations:** 1 Malawi Liverpool Wellcome Research Programme, Blantyre, Malawi; 2 Department of Clinical Sciences, Liverpool School of Tropical Medicine, Liverpool, UK; 3 Centre for TB Research, Liverpool School of Tropical Medicine, Liverpool, UK; 4 School of Medicine and Oral Health, Kamuzu University of Health Sciences, Blantyre, Malawi; 5 Clinical Research Department, London School of Hygiene & Tropical Medicine, London, UK; 6 School of Medicine, University of St Andrews, St Andrews, Scotland; 7 Helse Nord Clinical Research and Training Initiative, Kamuzu University of Health Sciences, Blantyre, Malawi; 8 Division of Epidemiology and Biostatistics, School of Public Health, Faculty of Health Sciences, University of the Witwatersrand, Johannesburg, South Africa; 9 TB Modelling Group, TB Centre, London School of Hygiene & Tropical Medicine, London, UK; 10 Department of Infectious Disease Epidemiology, London School of Hygiene & Tropical Medicine, London, UK; 11 Instituto de Medicina Tropical Alexander von Humboldt, Universidad Peruana Cayetano Heredia, Lima, Peru; 12 National Tuberculosis and Leprosy Elimination Program, Ministry of Health, Lilongwe, Malawi; 13 School of Health and Wellbeing, University of Glasgow, Glasgow, Scotland

**Keywords:** *Mycobacterium tuberculosis*, Infection, Immunoreactivity, Cross-sectional study, Sex, Adolescence, Risk factors, QuantiFERON-Gold TB Plus

## Abstract

**Background:**

Sex differences in *Mycobacterium tuberculosis* (Mtb) exposure likely contribute to sex differences in tuberculosis (TB) burden. However, population-level age- and sex-specific patterns of Mtb exposure among adolescents and adults have not been recently characterised in Malawi. We therefore conducted a cross-sectional survey to estimate age- and sex-specific Mtb immunoreactivity prevalence in Blantyre City, Malawi, and to assess whether prevalence diverges by sex with increasing age.

**Methods:**

We used an open-access dataset of building footprints, known as Open Buildings, to obtain a random sample of households from 33 peri-urban neighbourhoods in Blantyre City. Adolescents and adults aged 10–40 years were recruited, participant characteristics recorded, and venous blood samples collected to determine Mtb immunoreactivity using the QuantiFERON-TB Gold Plus (QFT-Plus) assay. We fitted Bayesian multilevel logistic regression models to estimate the association between age, sex, and other risk factors with Mtb immunoreactivity, and compared predicted immunoreactivity prevalence across age-sex strata.

**Results:**

Of 2833 participants, 40.0% (1133/2833) were male, the median age was 21 years (interquartile range: 16–28 years), and 8.7% (179/2047) self-reported living with HIV. Overall, 17.8% (503/2833) of participants were Mtb positive, Mtb immunoreactivity prevalence was 17.8% (95% credible interval [CrI]: 16.4–19.2%), corresponding to an annual risk of TB infection of 0.88% (95% CrI: 0.80%-0.95%). Prevalence was similar by sex among 10-19- and 30-40-year-olds. However, among 20-29-year-olds, prevalence was higher among males compared to females: 26.3% (95% CrI: 22.0%-30.8%) and 17.7% (95% CrI: 14.8%-20.8%), respectively. The annual risk of Mtb immunoreactivity conversion increased, on average, at a faster rate among males compared to females from age 10 years, peaking at 21 years, where it was 1.58 (95% CrI: 0.82–2.80) times higher among males compared to females. Tobacco smoking and alcohol drinking, more prevalent among males compared to females, were associated with increased immunoreactivity probability.

**Conclusions:**

Adolescence appears to be a period of vulnerability for Mtb exposure, during which sex-specific vulnerabilities begin to emerge. Identifying the biological and social drivers of these vulnerabilities could inform targeted strategies to reduce sex disparities in TB.

**Supplementary Information:**

The online version contains supplementary material available at 10.1186/s44263-026-00302-w.

## Background

Globally, tuberculosis (TB) affects more men than women [1]. Of the estimated 10.7 million people who developed TB in 2024, 54% were men (males ≥ 15 years old), 35% women, and 11% were children and adolescents < 15 years old [1]. Both TB incidence and prevalence increase with age, but trends differ by sex [1]. Men experience 1.7 times greater TB incidence, but delay seeking care up to 1.5 years longer than women, and subsequently account for the majority of transmission to men, women and children, due to longer duration of untreated infectious TB and assortative mixing. Men also experience greater exposure to *Mycobacterium tuberculosis* (Mtb), compared to women, from late adolescence, which likely contributes to sex differences in TB [2]. Understanding drivers of sex differences in Mtb exposure could contribute to reducing disparities that sustain TB incidence, morbidity and mortality in men, women and children.

Mtb exposure is a prerequisite for TB disease [3]. The risk of progression from infection to disease depends both on time since infection and on host immune status, and increases at the extremes of age, and in individuals with compromised immunity due to HIV infection and malnutrition [3]. Risk factors for TB disease have been described previously [1, 3]. In contrast, risk factors for Mtb exposure and infection are less well characterised. Understanding drivers of Mtb exposure is essential for interrupting transmission. Population-based Mtb immunoreactivity surveys, using immunological memory tests such as tuberculin skin tests (TSTs) and interferon gamma release assays (IGRAs), provide insights into Mtb exposure patterns [3]. A recent systematic review and meta-analysis of population-based Mtb immunoreactivity surveys showed increasing annual risk of Mtb immunoreactivity among young men compared to young women, starting from late adolescence [2]. 

Preventing TB in individuals exposed to Mtb, who are at risk of disease compared to those not exposed, is key to reducing incidence to meet TB elimination targets [4]. Currently, the mainstay is TB preventive treatment (TPT), and additionally, antiretroviral therapy among people with HIV [5]. Until recently, priority groups for TPT in high TB incidence settings were household TB contacts under 5 years old and people living with HIV [6]. Recent WHO guidance recommends TPT for all household TB contacts regardless of age and HIV status [6]. Expanded TPT eligibility, particularly with shorter-duration, more efficacious, low-toxicity regimens, is likely to improve coverage and further reduce TB incidence [7]. However, in high burden settings, the majority of transmission occurs outside households, and the contribution of household exposure to Mtb transmission reduces with age [8]. Novel vaccines, once routinely available, could further reduce TB incidence, morbidity and mortality by preventing Mtb infection and progression to disease among adolescents and adults, who account for the majority of TB incidence and mortality [9]. Age and sex-specific Mtb immunoreactivity prevalence estimates are needed to guide planning and targeted implementation of future Mtb interventions towards groups at highest risk of infection and progression to disease. In Malawi, a national TST conducted in school children aged 6 to 12 years estimated an ARTI of 1%.

To obtain Mtb immunoreactivity prevalence estimates and investigate sex differences across age, and associated individual-, household- and community-level factors, and to inform future Mtb infection studies and strategies, e.g. vaccination or preventive treatment, we conducted a population-based cross-sectional QuantiFERON-TB Gold Plus (QFT-Plus) IGRA survey among adolescents and adults 10–40 years old in Blantyre, Malawi, where TB remains a public health problem, but no age- and sex-specific population-representative estimates for Mtb immunoreactivity are available.

## Methods

### Study design and setting

Between 16th October 2022 and 23rd March 2024, we conducted a community-based, cross-sectional survey of Mtb immunoreactivity prevalence among adolescents and adults 10–40 years old, in randomly sampled households, across peri-urban neighbourhoods of Blantyre City, Malawi [10]. We focused on 10–40 year olds, to include the transition period from childhood to adulthood, when Mtb exposure and TB disease patterns change due to biological and sociobehavioural factors [2, 11]. 

Blantyre City, with an estimated total population of 900,000 people in 2024 extrapolated from the 2018 national census [12], is the second largest city in Malawi. Adult HIV prevalence was estimated at 14.2% in 2019 [13]. A 2019 survey, conducted in neighbourhoods accounting for 80% of Blantyre City residents, estimated prevalent microbiologically-confirmed adult pulmonary TB disease at approximately 150 per 100,000 population, representing an 80% reduction from the estimated 1,014 per 100,000 urban prevalence in the 2013 national prevalence survey [14, 15]. Despite recent progress, TB epidemiology in Blantyre City is likely still characterised by disparities, with poor neighbourhoods, which tend to be farther from clinics, experiencing underdiagnosis and therefore untreated disease [16, 17]. Additionally, TB prevalence and routine notifications are higher in men compared to women [14, 17]. Since 2011, details of all people registering for TB treatment in Blantyre City are recorded, including their household geographical positioning system (GPS) coordinates since 2015, in an enhanced surveillance system [18]. In 2015, 72 clusters were defined for a cluster-randomised trial of TB/HIV interventions [19]. Clusters were community health worker catchment areas, with approximately 4,000 adult residents per cluster [19]. 

In Malawi, TB and HIV care services are provided free of charge in public health facilities, although health system-related barriers, poverty and socioeconomic factors hinder access and effectiveness, which likely differentially affects men and women [20]. Neonatal Bacille Calmette Guerin (BCG) vaccination coverage exceeds 95% [21]. TB preventive treatment (TPT) is recommended for recent household TB contacts, with recently updated guidelines now including short-course regimens and additional recommendations for TPT for HIV negative recent household contacts older than 5 years, in line with global recommendations [22]. 

### Study procedures

We selected 33 high TB burden neighbourhoods (33 of the 72 clusters described), which accounted for approximately 60% of TB notifications in Blantyre City between 2016 and 2020 [10]. To obtain a population-representative sample, we randomly selected households in the 33 neighbourhoods, and included all eligible and consenting individuals in selected households. First, we obtained a random sample of possible household structures (i.e. dwellings) from selected neighbourhoods, using Open Buildings [23]—an open-access dataset of building footprints generated using satellite images and artificial intelligence-based algorithms—as our household sampling frame [24]. To determine the number of households to sample per neighbourhood, the neighbourhood-specific proportion of 10–40 year olds out of the study area total, obtained from constrained 2020 WorldPop estimates [25], was multiplied by the study target number of households. The target number of households was based on the average number of 10–40 year olds per household, determined from a previous survey [14] and the study pilot phase. We defined households and membership as individuals living together and sharing meals. Using GPS-enabled tablet computers, a study team of nurses and field research assistants located selected household locations to screen members for study eligibility.

Household members aged 10–40 years old were eligible for inclusion if present when the study team visited the household and provided informed consent. Our only exclusion criteria were unwillingness or inability to undergo informed consent. Households were visited up to 2 times, at least once during the weekend, if eligible members were not present during the first study team visit. We screened additional households from the household sampling list until the neighbourhood age- and sex-specific recruitment target was met.

In households with at least one enrolled member, we recorded household GPS coordinates, household composition, head of household’s highest education level, access to potable water and sanitation facilities, previous TB treatment history, and asset ownership, using a household questionnaire based on the Malawi Integrated Household Indicator Survey [26]. We recorded participant age, sex, self-reported HIV status, past TB treatment, household TB contact history, any lifetime tobacco smoking and alcohol drinking, using a standardised questionnaire used in previous TB studies [10]. We defined household TB contact as sharing a household with an individual treated for TB. A trained nurse then obtained a 5 mL venous blood sample for QuantiFERON-TB Gold Plus (QFT-Plus) testing.

### Laboratory procedures

We followed QFT-Plus manufacturer’s instructions for blood sample handling and processing [27]. Blood samples were collected into 6mL lithium heparin tubes, transported at room temperature to a research laboratory within 6–8 h of collection, transferred into QFT-Plus nil, TB1, TB2 and mitogen tubes and incubated at 37 °C for 16–20 h. Following incubation, plasma was harvested, stored at -20 °C for enzyme-linked immunosorbent assay batch-processing up to 2 weeks later.

### Study outcome and covariate definition

The study primary outcome was Mtb immunoreactivity, i.e. a positive QFT-Plus result, defined as nil tube-subtracted TB1 or TB2 interferon gamma value ≥ 0.35 IU/ml, as per manufacturer instructions, calculated from the QFT-Plus software version 2.72 [27]. Participant age (integer years) was categorised into three groups (10–19, 20–29 and 30–40 years) for summaries; models used both categorised and integer values. Most covariates were recorded as binary or categorical responses: sex (male/female), HIV status (positive/negative/unknown), previous TB treatment or household TB contact (yes/no). Tobacco smoking and alcohol drinking were recoded into “past/current” (i.e. any lifetime history), and “never”. Unknown values were treated as missing. We calculated a household poverty score as a proxy for socio-economic status, using household variables as calculated previously [38]. We obtained 100m^2^ gridded study area population count estimates for 2020 from WorldPop [25], used the sf R package v1 1.0.16 [28], to extract study geographical area estimates from shapefiles defined previously [19], and then estimated neighbourhood population densities. We acquired neighbourhood-level adult HIV prevalence estimates, modelled previously through spatio-temporally explicit Bayesian models using data from a 2019 neighbourhood survey and a 2020 national survey [29, 30]. We calculated neighbourhood-level TB case notification rates (CNR) for 2022 using notifications from the enhanced surveillance system described above, and population denominators from WorldPop population estimates [18]. 

## Management of participants with positive QFT-Plus test results

In Malawi, TB preventive treatment is recommended for recent household TB contacts and people initiating treatment for HIV infection [22], as described above. Testing positive for Mtb immunoreactivity is not an indication for treatment or investigation for TB disease [45]. The unknown timing and typically historical nature of exposure among general community adolescents and adults testing positive on Mtb immunoreactivity tests, and low risk of incident disease among remotely infected individuals, make the risk-benefit assessment of treatment in the absence of other risk factors uncertain [5]. Therefore, QFT-Plus positive participants were not treated or investigated further in this study. Nevertheless, at enrolment, all participants were given general TB and HIV advice and encouraged to seek care in case of TB symptoms, with messages repeated when communicating QFT-Plus results [10]. 

### Sample size

Our primary objective was to estimate age- and sex-specific Mtb immunoreactivity prevalence and respective male-to-female (M:F) ratios. Based on pilot work and previous survey estimates [31], we assumed that Mtb immunoreactivity prevalence would exceed 20% from age 10 years, and be higher among males than females. Fixing power at ≥ 80% and two-sided α = 0.05, and assuming 20% prevalence in females, recruiting a total of 3,652 participants, in three age groups of unequal size but equal recruitment by sex, would be sufficient to detect M:F prevalence ratios ≥ 1.35 [10]. We did not account for clustering of Mtb immunoreactivity at the household or neighbourhood level a priori due to lack of setting-specific intra-class correlation estimates for QFT-Plus positivity. Details are in the published protocol [10]. 

### Statistical analysis

We summarised participant characteristics using medians and interquartile ranges (IQR) or counts and proportions by age and sex. We estimated age- and sex-specific Mtb immunoreactivity prevalence as the proportion of QFT-Plus positive individuals, excluding those with indeterminate results. Using conjugate beta prior distributions for the binomial likelihood, we represented uncertainty using 95% Bayesian credible intervals (CrIs) derived from the posterior beta distributions. For the beta prior distributions, we used weakly informative Kerman priors [32]. We calculated the corresponding annual risk of TB infection (ARTI) using the formula: ARTI = 1-(1-P)^1/mean age^ [33], where P is the estimated Mtb immunoreactivity prevalence, with 95% CrIs propagating uncertainty from the prevalence estimates. To explore the potential effect of IGRA reversion on the estimated ARTI [34], we multiplied our “naïve” ARTI estimates by a median true: naïve ARTI ratio, calculated using Schwalb et al.’s method [35]: median ratio 2.82 (range 1.52–4.74), assuming IGRA reversion rates ranged between 8.8% and 11.1%, based on an IGRA survey of 12 to 18 year olds from South Africa [35, 36]. 

We used multilevel Bayesian logistic regression models to estimate (1) age- and sex-specific odds of Mtb immunoreactivity, and (2) odds ratios of individual-, household- and neighbourhood-level covariates. We used published TB epidemiology literature to guide initial covariate selection [3, 37, 38], and used leave-one-out cross-validation [39] for selecting final model covariate sets (Supplementary Material 1: Fig. S1). To address our primary objective—investigating sex differences in Mtb immunoreactivity across three age groups, as prespecified [10]—we first fitted a model with age categorised into three groups: 10–19, 20–29, and 30–40 years, and included an interaction with sex to estimate age- and sex-specific odds of Mtb immunoreactivity.

We then examined finer age patterns using a “base model” in which age was modelled as an integer via a thin-plate regression spline function, specified with an offset for sex. The “fully adjusted model” added HIV status, previous TB treatment, household TB contact, tobacco smoking, and alcohol drinking to the base model to estimate age- and sex-adjusted associations. We also assessed household poverty and neighbourhood-level variables (TB case notification rates [CNRs], adult HIV prevalence, and population density), adjusting for all individual-level covariates. TB CNRs and population density were mean-centred to improve sampling efficiency. All models included a neighbourhood-level random effect, to account for the cluster-based household sampling. We used complete case analysis. Priors were weakly informative, with the influence of stronger and weaker priors on posterior inferences visualised via density plots from the priorsense R package v1.1.0 [40], which uses prior and likelihood power scaling to explore posterior sensitivity.

We predicted age- and sex-specific Mtb immunoreactivity probability from the age- and sex-adjusted model to explore changes in immunoreactivity risk with age, excluding neighbourhood random effects. The annual risk of Mtb immunoreactivity conversion experienced at each year of age was calculated as the change in predicted immunoreactivity probability between consecutive ages, divided by 1-immunoreactivity probability (i.e. proportion still at risk of converting from negative to positive immunoreactivity status) at the initial age, expressed as a percentage, following Middelkoop et al. [41] See Supplementary Material 1: Section B. We calculated age-specific male-to-female (M:F) ratios from predicted probabilities and annual risk of immunoreactivity conversion to quantify the relative risk of Mtb immunoreactivity comparing males to females. The posterior probability of excess male Mtb immunoreactivity risk (i.e. M:F ratio > 1) was computed as the proportion of posterior draws where M:F ratio exceeded 1, expressed as a percentage and associated Monte Carlo standard error. We used the “full” model to predict probabilities of Mtb immunoreactivity and M:F ratios across combinations of covariates to explore the implications of model-estimated odds ratios on immunoreactivity probability. In a sensitivity analysis, we recalculated age-specific immunoreactivity M:F ratios using predictions from the “base” model refitted to data for HIV negative individuals only, to explore the influence of HIV prevalence on the trajectories of M:F ratios.

Models were fitted in the R environment for statistical computing [42] (v4.3.3), using the brms package v2.22.0 [43], an interface for implementing multilevel Bayesian models and probabilistic parameter sampling in Stan [44]. Inferences were based on 4,000 post-warm up posterior samples from 4 parallel Markov chain Monte Carlo (MCMC) chains. We visualised posterior distributions using line and density plots, and used means and 95% central percentiles as credible intervals for summaries. We inspected MCMC convergence via trace plots, Gelman-Rubin’s potential scale reduction factor statistic and effective sample sizes, and assessed model fit using posterior predictive checks. See Supplementary Material 1: Section B.

## Results

### Participant characteristics and QFT-Plus positivity results

We enumerated 8031 individuals of whom 65.4% (5259/8031) were age-eligible. Of age-eligible individuals, 33.4% (1759/5,259) were unavailable, and 2.7% (140/5259) refused screening. Of eligible individuals approached for informed consent, 10.2% (344/3360) declined, the majority of whom (65.1% [224/344]) indicated “do not want to participate in research”. We enrolled 89.8% (3016/3360) of those approached for informed consent, from 1436 households, with a median of 2 (range: 1–11) people enrolled per household. We included 93.9% (2833) of the enrolled participants with a positive or negative QFT-Plus result in the analysis, excluding 2.1% (62/3016) of participants with an indeterminate result, and 4.0% (121) of participants with insufficient blood samples, processing error or missing test results (Supplementary Material 1: Fig. S2).

Overall, participants’ median age was 21 years (interquartile range: 16–28 years) and 40.0% (1133/2833) were male (Table 1). We enrolled fewer males than females in older age groups: 47.1% (578/1,228) males among 10–19 year olds versus 29.2% (174/595) among 30–40 year olds (Table 1). Overall, 8.7% (179/2047) self-reported being HIV positive, of whom 97.8% (175/179) self-reported being on antiretroviral therapy. Among adults, the proportion self-reporting being HIV positive was higher among women compared to men of the same age: 1.9% (6/316) males and 6.8% (40/590) females, among 20–29 year olds, and nearly twice in females compared to males among 30–40 year olds: (11.5% [18/157] males and 22.2% [93/418] females) (Table 1). Overall, 1.4% (40/2,821) of participants reported previous TB treatment, with roughly similar proportions by sex across age groups: overall 1.6% (18/1,129) among males and 1.3% (22/1,692) among females. Overall, 10.7% (301/2,804) reported previous household TB contact, with roughly similar proportions by sex. The proportion reporting cough ≥2 weeks, any TB symptoms, and those categorised as residing in the poorest third of households did not vary by sex (Table 1).

**Table 1:** Participant characteristics, QuantiFERON-Plus Gold (QFT-Plus) results, Mycobacterium immunoreactivity prevalence, and annual risk of tuberculosis infection (ARTI) by age and sex

Characteristics	Overall	Age group and sex					
**Age group (years)**		10–19 years		20–29 years		30–40 years	
% (n)	**100% (2833)**	**43.3% (1228)**		**35.7% (1010)**		**21% (595)**	
**Sex**		**Male**	**Female**	**Male**	**Female**	**Male**	**Female**
% (n)	40.0% (1133/2833)	**47.1% (578)**	**52.9% (650)**	**37.7% (381)**	**62.3% (629)**	**29.2% (174)**	**70.8% (421)**
Age years median (IQR)	21 (16–28)	15 (12–17)	15 (12–18)	23 (21–26)	24 (22–26)	35 (32-37.8)	35 (32–38)
HIV positive (self-reported)	8.7% (179/2047)	4.3% (11/254)	3.5% (11/312)	1.9% (6/316)	6.8% (40/590)	11.5% (18/157)	22.2% (93/418)
Cough ≥ 2 weeks	1.1% (32/2833)	1.2% (7/578)	0.8% (5/650)	1.3% (5/381)	1.3% (8/629)	2.9% (5/174)	0.5% (2/421)
Any TB symptoms	14% (396/2833)	12.6% (73/578)	15.8% (103/650)	12.9% (49/381)	12.4% (78/629)	16.7% (29/174)	15.2% (64/421)
Previous TB treatment^1^	1.4% (40/2821)	0.5% (3/575)	0.5% (3/647)	2.1% (8/381)	0.8% (5/625)	4.0% (7/173)	3.3% (14/420)
Previous Household TB contact^1^	10.7% (301/2804)	12.7% (72/497)	11.7% (75/568)	9.2% (35/346)	8.7% (54/566)	10.3% (18/156)	11.3% (47/370)
Past/current tobacco^1^ smoker	13.3% (376/2829)	13% (75/503)	3.2% (21/628)	37.9% (144/236)	6.5% (41/587)	42.0% (73/101)	5.2% (22/398)
Past/current alcohol drinking^1^	28.6% (810/2829)	22% (127/578)	10.8% (70/578)	66.9% (255/381)	22.5% (141/627)	75.3% (131/174)	20.4% (86/421)
Household in poorest quartile	26% (735/2830)	26.5% (153/424)	26% (169/480)	27.6% (105/276)	27.7% (174/454)	19.5% (34/140)	23.8% (100/321)
QFT-Plus^2^ positive	17.8% (503/2833)	11.4% (66/512)	10.6% (69/581)	26.2% (100/281)	17.6% (111/518)	29.3% (51/123)	25.2% (106/315)
Mtb immunoreactivity prevalence^3^% (95% CrI)^4^	17.8% (16.4%-19.2%)	11.5% (9.0%-14.2%)	10.7% (8.4%-13.1%)	26.3% (22.0%-30.8%)	17.7% (14.8%-20.8%)	29.4% (22.9%-36.3%)	25.2% (21.2%-29.5%)
ARTI^5^% (95% CrI)	0.88% (0.80%-0.95%)	0.82% (0.64%-1.04%)	0.75% (0.58%-0.94%)	1.29% (1.05%-1.56%)	0.81% (0.66%-0.96%)	1.00% (0.75%-1.29%)	0.83% (0.68-1.00%)

^1^Overall total number of participants, some rows have smaller denominators due to missing/unknown values: 786 participants reported unknown HIV status, 12 had missing TB treatment history, 29 had missing household TB contact (defined as living in the same household with a person receiving tuberculosis treatment. ^2^QFT-Plus: QuantiFERON-Plus assay. ^3^*Mycobacterium tuberculosis* (Mtb) immunoreactivity was defined as a positive QFT-Plus test result (cut-off ≥ 0.35 IU/mL). ^4^95% CrI: 95% credible intervals. ^5^ARTI: Annual Risk of TB Infection, calculated from Mtb immunoreactivity prevalence estimates, not adjusted for reversion, using the formula: ARTI = 1-(1-P)^1/mean age^

The proportion of participants who reported any lifetime tobacco smoking or alcohol drinking varied by sex. Overall, 13.3% (376/2,829) reported any lifetime tobacco smoking. Among 10–19 year olds, 13% (75/503) among males and 3.2% (21/628) among females reported lifetime tobacco smoking. Among 30–40 year olds, the proportion of any lifetime tobacco smoking history among males was 8 times that in females: 42% (73/101) compared to 5.2% (22/398), among males and females, respectively. Overall, 28.6% (810/2,829) reported any lifetime alcohol drinking. Compared to females (20.4% [86/421]), 3.7 times more males (75.3% [131/174]) reported any lifetime alcohol drinking among 30–40 year olds (Table 1).

In total, 503/2,833 (17.8%) participants were Mtb immunoreactive, i.e. QFT-Plus positive: 19.2% (217/1,133) among males and 16.8% (286/1,700) among females.

### Mtb immunoreactivity prevalence and annual risk of TB infection (ARTI)

Overall, the estimated Mtb immunoreactivity prevalence was 17.8% (95% credible interval [CrI]: 16.4%-19.2%), and increased with age among both males and females, but with sex divergence. Whereas prevalence was similar by sex among 10–19 year olds: 11.5% (95% CrI: 9.0%-14.2%) among males, and 10.7% (95% CrI: 8.4%-13.1%) among females, prevalence was higher among males than females in 20–29 year olds: 26.3% (95% CrI: 22.0%-30.8%) among males and 17.7% (95% CrI: 14.8%-20.8%) among females. Among 30–40 year olds, prevalence was 29.4% (95% CrI: 22.9%-36.3%) and 25.2% (95% CrI: 21.2%-29.5%) among males and females, respectively. Corresponding ARTI estimates were: 0.88% (95% CrI: 0.80%-0.95%) overall, and highest among males aged 20–29 years (95% CrI: 1.24% [0.87%-1.69%]) (Table 1). When multiplied by the median true: naïve ARTI ratio (2.82), ARTI estimates were 2.48% (95% CrI: 2.26%-2.68%) overall, and 3.64% (95% CrI: 2.96%-4.40%) among males aged 20–29 years.

### Mtb immunoreactivity and associated factors

#### Age and sex

**Fig. 1:**
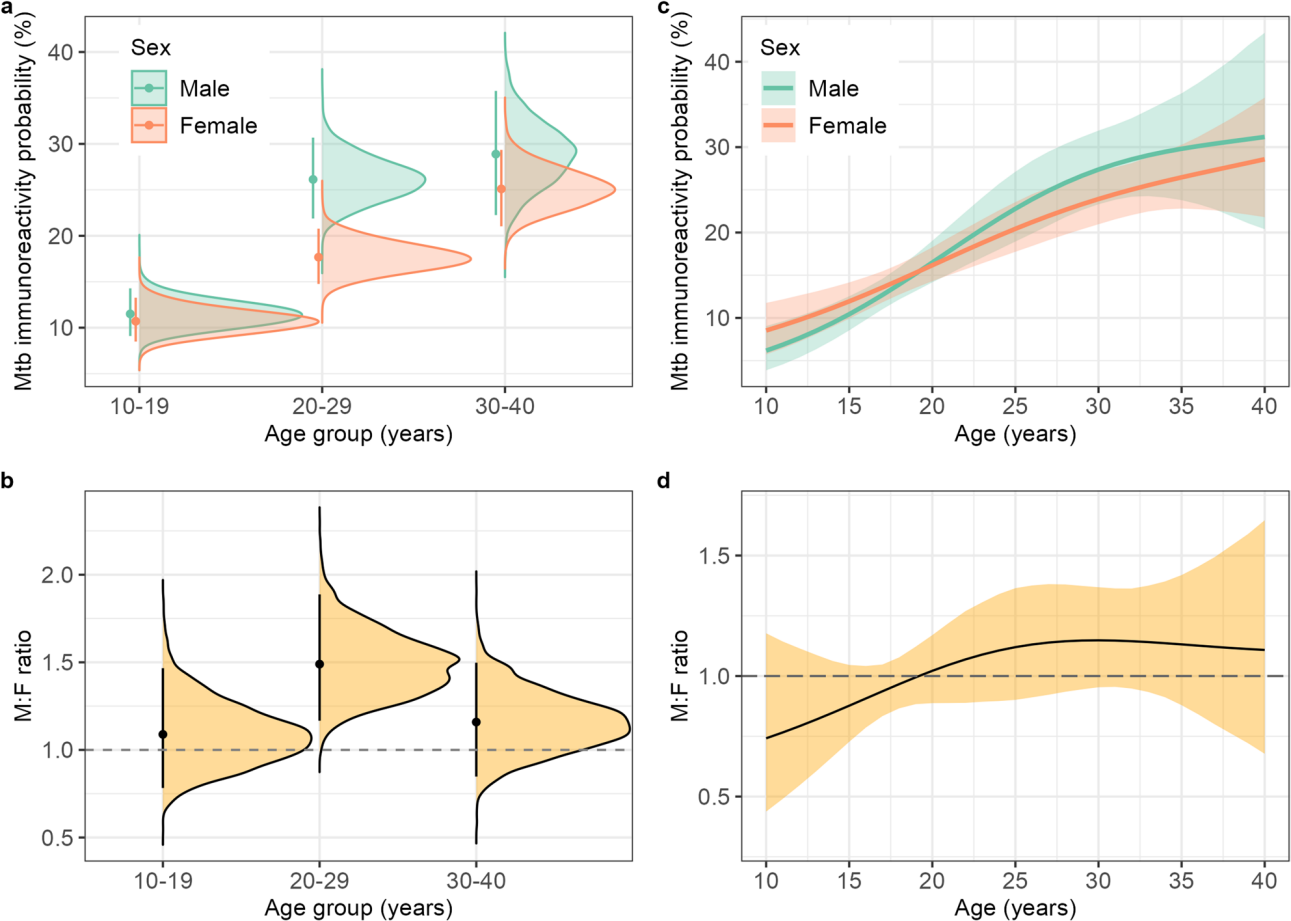
Density plots and line graphs of predicted *Mycobacterium tuberculosis* (Mtb) immunoreactivity probability by age and sex (top row), and corresponding male-to-female (M:F) ratios by age (bottom row). Age was fitted as a categorical covariate, with an interaction with sex (panel **A**), and as an integer covariate via a thin-plate regression spline function, with an offset for sex (panel **C**). Probabilities are exponentiated odds predicted from a Bayesian logistic regression model of Mtb immunoreactivity, adjusted for age and sex. Mtb immunoreactivity was defined as a positive QuantiFERON-TB Gold Plus test result. In panels **a** and **b**, points are posterior means, with lines representing associated 95% credible intervals. Shaded areas are densities. In panels **c** and **d**, solid lines are posterior means, and shaded areas are 95% credible intervals. The dashed lines in panels **b** and **d**, M:F ratio = 1, represent equal immunoreactivity probabilities between males and females The estimates for predicted immunoreactivity probability and corresponding M:F ratios (i.e. panels **a** and **b** versus **c** and **d**) do not align perfectly because, although fitted to the same data, estimates are from different models

Overall, the probability of Mtb immunoreactivity, predicted from models adjusted for age and sex, increased with age among both males and females (Fig. 1). When age was modelled as categorical variable, the predicted immunoreactivity probability was similar by sex among 10–19 year olds: 11.5% (95 CrI: 9.1%-14.3%) and 10.7% (8.5%-13.3%) among males and females, respectively, with a male-to-female (M:F) ratio of 1.09 (95% CrI: 0.78–1.47). However, among 20–29 year olds, immunoreactivity probability was higher among males compared to females: 26.1% (21.9%-30.7%) and 17.7% (14.8%-20.8%), respectively, with a M:F ratio of 1.49 (95% CrI: 1.17–1.89). Among 30–40 year olds, immunoreactivity probability was 28.9% (22.3%-35.8%) and 25.1% (21%-29.3%). The corresponding M:F ratio was 1.16 (0.85–1.5) (Fig. 1). Models fitted with age specified as a categorical covariate demonstrated poor fit to the data (Supplementary Material 1: Section G).

Modelling age as an integer revealed diverging Mtb immunoreactivity probability age trends by sex from late adolescence. Before age 20 years, females had, on average, higher mean predicted Mtb immunoreactivity probability compared to males, but 95% CrIs overlapped. At age 10 years, the predicted Mtb immunoreactivity probability was 6.2% (95% CrI: 3.9%-9.1%) in males and 8.5% (95% CrI: 5.7%-11.8%) in females, with M:F ratio 0.74 (95% CrI: 0.44–1.18) (Supplementary Material 1: Table S1). In contrast, from age 20 years, males had, on average, higher predicted Mtb immunoreactivity probability compared to females: 16.5% (95% CrI: 14.2%-19.0%) in males and 16.2% (95% CrI: 14.3%-18.3%) in females at age 20 years, and 31.2% (95% CrI: 20.4%-43.4%) in males and 28.6% (95% CrI: 21.8%-35.8%) in females at age 40 years. Corresponding M:F ratios were 1.02 (95% CrI: 0.89–1.17) and 1.11 (95% CrI: 0.68–1.65) at ages 20 and 40 years, respectively. The M:F ratio peaked at ages 29 to 31 years, where it was 1.15 (95% CrI: 0.94–1.37) among 29 year olds (Supplementary Material 1: Table S1).

The posterior probability of excess male Mtb immunoreactivity risk, i.e. the proportion of posterior draws where the M:F ratio exceeded 1, rose from 9.9% at age 10 years to 45.8% at age 19 years. From age 20 years, the posterior probability of excess male risk was ≥ 60%, and was approximately 90% between ages 29 and 33 years (Supplementary Material 1: Table S1).

**Fig. 2:**
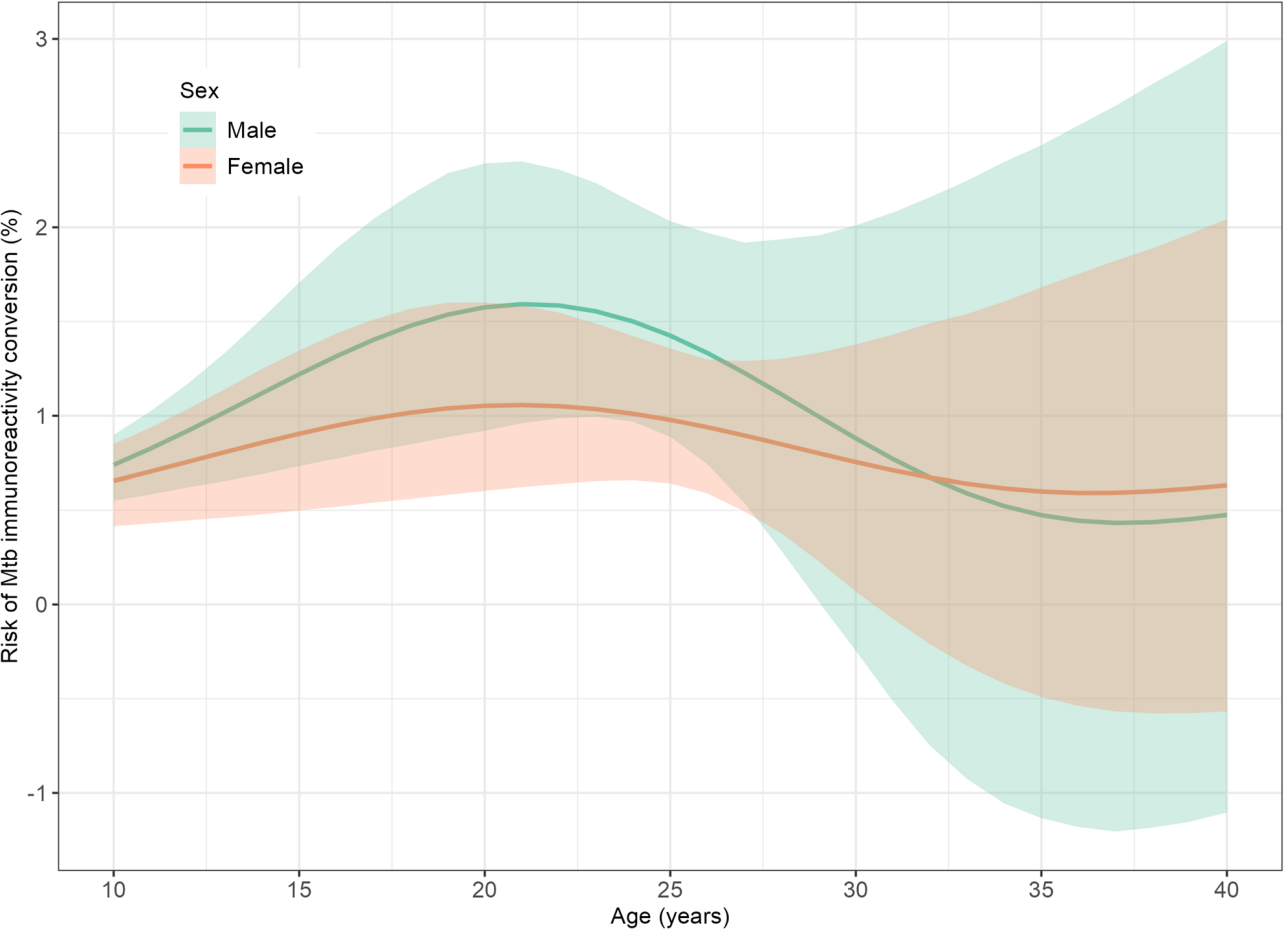
Risk of *Mycobacterium tuberculosis* (Mtb) immunoreactivity probability by age and sex. The risk of immunoreactivity conversion at each year was calculated as the annual change in predicted Mtb immunoreactivity probability by the next year of age, divided by 1-immunoreactivity probability (i.e. proportion still at risk of converting from negative to positive immunoreactivity status) at that given year. Mtb immunoreactivity was defined as a positive QuantiFERON-TB Gold Plus test result. Solid lines are posterior means, shaded areas are 95% credible intervals. Risk of conversion estimates included negative values at older ages, reflecting increasing uncertainty in predicted Mtb immunoreactivity probabilities

The risk of converting from negative to positive Mtb immunoreactivity status increased with each additional year of age, before declining from the early 20s onward. The annual risk of conversion to Mtb immunoreactivity increased from age 10 years, peaking at 21 years among both males and females: 1.59% (95% CrI: 0.96%-2.35%) and 1.03% (95% CrI: 0.6%-1.6%), among males and females, respectively (Fig. 2). These annual risk of conversion estimates corresponded to a 1.58 (95% CrI: 0.82–2.80) times higher risk of conversion among males compared to females at the peak age (21 years). Overall, males aged 21 years experienced the greatest increase in annual risk of immunoreactivity conversion (Fig. 1). At older ages, the annual risk of conversion declined, and the corresponding 95% CrIs included negative values despite persistently increasing mean predicted immunoreactivity probabilities among both males and females, reflecting increasing uncertainty in predicted immunoreactivity probabilities (Fig. 2).

#### Other individual-level factors

After adjusting for age and sex, HIV status was not associated with Mtb immunoreactivity: age- and sex-adjusted odds ratio (OR) 0.79 (95% CrI: 0.53–1.16) (Table 2). Previous TB treatment had the largest odds ratio for immunoreactivity, with strong evidence of the association: OR 2.60 (95% CrI: 1.40–4.82), while the OR for previous household TB contact was 1.44 (95% CrI: 1.06–1.93) (Table 2). There was strong evidence that any lifetime tobacco smoking and alcohol drinking were associated with increased odds of immunoreactivity, ORs 1.54 (95% CrI: 1.18-2.00 and 1.53 (95% CrI: 1.23–1.91), respectively. When simultaneously adjusted for all other individual covariates, the magnitude of associations reduced slightly, but the direction remained unchanged (Table 2). However, the evidence of association for previous household TB contact and tobacco smoking weakened substantially. Notably, 786 participants were excluded from multivariable analysis adjusted for HIV due to unknown status: 84% (622/786) were adolescents 10–19 years old, 99% (655) of whom had not previously tested for HIV.

**Table 2:** Factors associated with Mycobacterium tuberculosis immunoreactivity from regression models* adjusted for age and sex

Covariate	QFT-Plus^1^ positive	Age & sex-adjusted OR^2*^ (95% CrI)		Fully adjusted OR^3*^ (95% CrI)
**Individual-level covariates**				
HIV status				
Negative	20.1% (376/1868)		Ref	
Positive	20.1% (36/179)		0.79 (0.53–1.16)	0.70 (0.46–1.04)
Previous TB treatment				
No	17.3% (482/2781)		Ref	
Yes	45.0% (18/40)		2.60 (1.40–4.82)	2.17 (1.09–4.35)
Previous household TB contact^4^				
No	17.3% (434/2503)		Ref	
Yes	22.6% (68/301)		1.44 (1.06–1.93)	1.29 (0.89–1.85)
Tobacco smoking				
Never	16.4% (402/2453)		Ref	
Past/current	26.6% (100/376)		1.54 (1.18-2.00)	1.24 (0.88–1.72)
Alcohol drinking				
Never	15.0% (302/2019)		Ref	
Past/current	24.8% (201/810)		1.53 (1.23–1.91)	1.40 (1.08–1.83)
**Household and neighbourhood-level covariates**				
Household in poorest quartile			1.05 (0.82–1.35)	1.06 (0.84–1.37)
Neighbourhood TB case notification rate^5^, per additional ~ 150/100,000 notifications			1.11 (0.98–1.25)	1.02 (0.90–1.17)
Neighbourhood adult HIV prevalence, per 1% increase^5^			0.67 (0.10–4.49)	0.84 (0.13–5.77)
Neighbourhood population density^5^, per additional ~ 2,000 people per km^2^			1.18 (1.05–1.33)	1.16 (1.01–1.33)

^1^QFT-Plus: QuantiFERON-TB Gold Plus test. ^2^Age- and sex-adjusted OR: Odds ratio and 95% credible interval. For individual-level covariates, the odds ratio was estimated from bivariate models additionally adjusted for age and sex. For household and neighbourhood-level covariates, odds ratios were from bivariate models additionally adjusted for age and sex, and for all individual-level effects included in the table. ^3^Fully adjusted OR: Odds ratio and 95% credible interval. For individual-level covariates, odds ratios were adjusted all individual-level covariates, while odds ratios for household- and neighbourhood-level covariates were adjusted for all individual-, household-, and neighbourhood-level covariates included in the table. ^4^Previous household TB contact: Household tuberculosis (TB) contact was defined as sharing a household with a person treated for TB. ^5^ Covariate was standardised (mean subtracted and divided by the mean) before model fitting. Odds ratios, therefore, represent the change in odds per standard deviation increase in the covariate. Standard deviations were 159 case notifications per 100,000 population for TB case notification rate, and 2,010 people per km^2^ for population density. *Estimates are from multilevel Bayesian regression models adjusted for age specified as a spline term, with an offset for sex, including a neighbourhood level random effect. Models were fitted to different sets of observations due to missing data: 2,833 observations for models adjusted for age and sex only, and 2014 observations for models adjusted for all individual and neighbourhood level covariates. The neighbourhood random intercept standard deviation (log-odds scale) was 0.133 (95% CrI: 0.006–0.324) from the model adjusted for individual-level variables only, and 0.104 (95% CrI: 0.005–0.269) for the model additionally adjusted for neighbourhood covariates

#### Household and neighbourhood factors

After adjusting for all individual covariates, there was moderate evidence that neighbourhood population density was associated with increased odds of immunoreactivity, OR 1.18 (95% CrI: 1.05–1.33) (Table 2). The evidence for association for household poverty, neighbourhood TB case notification rate (CNR) and adult HIV prevalence was weak (Table 2). However, some unexplained neighbourhood heterogeneity remained even after adjusting for neighbourhood-level population density (Table 2).

### Comparing predicted Mtb immunoreactivity probability across combinations of risk factor levels

**Fig. 3:**
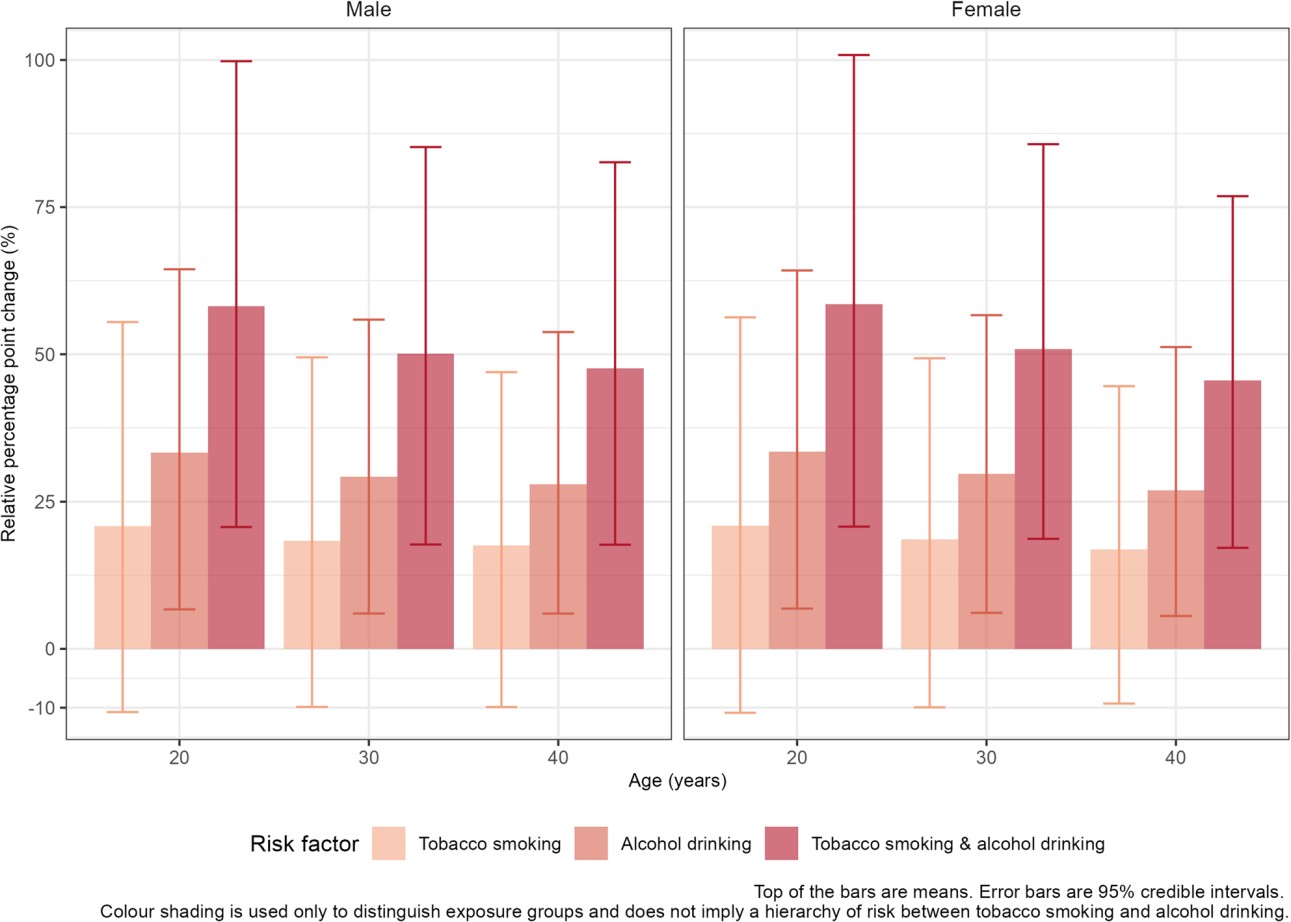
Relative percentage point change in predicted *Mycobacterium tuberculosis* (Mtb) immunoreactivity probability comparing individuals with any lifetime alcohol drinking or tobacco smoking history at selected ages by sex. The baseline group was HIV negative individuals without previous TB treatment or household TB contact history, without any lifetime history of alcohol drinking or tobacco smoking. Probabilities were exponentiated odds of Mtb immunoreactivity estimated from a Bayesian logistical regression model adjusted for age, sex, HIV status, previous TB treatment history, household TB contact history, any lifetime alcohol drinking and any lifetime tobacco smoking. Mtb immunoreactivity was defined as a positive QuantiFERON-TB Gold Plus test. HIV status was self-reported. The relative percentage point change was calculated as the predicted probability in the comparison group minus the baseline group, divided by the baseline group x 100. Top of the bars are means. Error bars are 95% credible intervals

Consistent with model-estimated effects above, any lifetime alcohol drinking and/or tobacco smoking increased, on average, the predicted probability of Mtb immunoreactivity (Fig. 3). Compared to a baseline group of HIV negative individuals not previously treated for TB and without household TB contact history, any lifetime alcohol drinking and tobacco smoking were associated with increased predicted probability of Mtb immunoreactivity. Among 20 year olds, the predicted immunoreactivity probabilities were: 14.3% (11.8%-17%) in the baseline group and 19.0% (15.0%-23.7%) among individuals with any lifetime history of alcohol drinking, with a relative percentage point increase of 33.3% (6.8%-64.3%). Among 40 year olds, the predicted immunoreactivity probability was 25.9% (16.4%-35.2%) in the baseline group, and 32.8% (21.1%-44.4%) among individuals with any lifetime history of alcohol drinking, with a relative percentage point increase of 27.4% (5.7%-52.6%) (Fig. 3). The relative changes in predicted probabilities were similar among males and females, as expected, as models did not include an interaction between sex and alcohol drinking or tobacco smoking (Fig. 3). Models which included an interaction between sex and alcohol drinking and tobacco smoking, respectively, did not provide conclusive evidence of differences in the association between Mtb immunoreactivity and alcohol drinking and tobacco smoking by sex. See Supplementary Material 1: Fig. S3.

### Sensitivity analysis

When recalculated using predictions from the age- and sex-adjusted model fitted to data for HIV negative individuals only, age-specific M:F ratios of Mtb immunoreactivity probability and the posterior probability of male excess were broadly similar throughout adolescence to estimates from models fitted to data from all participants. However, the posterior probabilities of excess male risk reduced quickly from 30 years in models fitted to data from HIV negative individuals only, compared to models fitted to all participants (Supplementary Material 1: Fig. S4). Model inferences were robust in terms of prior specification (Supplementary Material 1: Fig. S5). Full details of regression coefficients from fitted models and model convergence characteristics are in Supplementary Material 1: Section H.

## Discussion

In this first community-based Mtb immunoreactivity survey among adolescents and adults aged 10–40 years old in Blantyre, Malawi, we found similar immunoreactivity prevalence and ARTI by sex among 10–19 year olds. However, among 20–29 year olds, prevalence and ARTI were higher among men compared to women, but converged among 30–40 year olds. From age 10 years, the annual risk of Mtb immunoreactivity conversion increased at a faster rate in males compared to females, before declining among both males and females from the early 20s, and plateauing later in adulthood. Additional risk factors for Mtb immunoreactivity included any lifetime alcohol drinking and tobacco smoking history, substantially more prevalent among men than women, and residence in densely populated neighbourhoods. Our findings suggest that adolescence is a period when age-specific trajectories of Mtb exposure and infection risk diverge by sex, with important implications for TB prevention and care strategies in urban African settings.

Our findings are consistent with a recent systematic review and meta-analysis of 81 population-representative Mtb immunoreactivity surveys conducted between 1993 and 2022 across 38 countries, which included approximately 500,000 individuals and showed a higher prevalence of Mtb immunoreactivity among young men than young women starting from late adolescence [2]. The annual risk of Mtb immunoreactivity conversion in men was 1.38 times that in women at age 20 years, increasing to 1.52 times by age 50 years [2]. These differences were consistent over time, geographical settings, study assay (TST or IGRA), and country TB incidence [2]. Our survey similarly found male-to-female (M:F) ratios of annual risk of immunoreactivity conversion exceeding one from early adulthood, reinforcing evidence that sex disparities in Mtb exposure and susceptibility begin to diverge in adolescence. Notably, in our study, this sex divergence peaked approximately a decade earlier than reported in the systematic review, suggesting that male vulnerability to infection arises sooner in this study population, compared to populations in surveys included in the meta-analysis [2]. Furthermore, this survey, in contrast to many others before, is from a high HIV prevalence urban setting with high rates of urbanisation and therefore likely different risk behaviours compared to rural areas.

Our main hypothesis is that boys and young men experience greater exposure to Mtb than girls and young women, therefore a higher rate of immunoreactivity conversion, due to changing social contact patterns. During adolescence, increasing social contacts, e.g. in schools, public transport, and other crowded environments, and workplaces later on in adulthood, increase opportunities for Mtb exposure [46, 47]. In many settings, boys and young men spend more time in congregate social spaces such as bars and video showrooms, which are conducive for airborne transmission [48]. Increasing contacts with older men, who are likely to have infectious TB [49, 50], further amplifies Mtb exposure risk. Tobacco smoking and alcohol drinking, more prevalent among men than women in our study, and likely proxies for other shared sociobehavioural risk factors, were associated with increased Mtb immunoreactivity. Therefore, increasing exposure, due to increasing infectious TB social contacts starting from adolescence, is a more likely explanation for the faster increase in Mtb immunoreactivity prevalence among boys and young men compared to girls and young women.

Biological differences between males and females could also partly explain the sex differences in Mtb immunoreactivity observed in our study. Sex-related immunological changes may heighten susceptibility or reduce containment of Mtb among males. The role of sex-specific immune pathways as likely contributors to the higher male burden of Mtb infection and disease has been previously debated [51]. Our study did not explore biological mechanisms. However, it is plausible that biological factors act in concert with sociobehavioural determinants, amplifying male vulnerability to Mtb infection.

Beyond individual- and sex-specific determinants, structural and environmental conditions at the community level play a key role in shaping Mtb exposure patterns. In our study, neighbourhood population density was associated with higher Mtb immunoreactivity risk in multivariate analysis. This association persisted after adjusting for neighbourhood TB notification rates, adult HIV prevalence, and household poverty. In informal peri-urban settlements, high population density often reflects overcrowding and poor housing, conditions that facilitate transmission through prolonged indoor exposure and limited ventilation [52]. The absence of association between Mtb immunoreactivity and neighbourhood TB notification rate likely reflects the limitations of using routine surveillance data as a proxy for transmission intensity. Notifications are an imperfect measure of prevalent infectious TB, i.e. the force of infection, due to delayed detection, incomplete case finding, and variable care-seeking [53]. Together, these findings highlight the role of structural and environmental conditions, rather than TB incidence alone, in sustaining TB transmission risk in informal urban communities.

This study has several limitations. First, statistical power to detect M:F immunoreactivity probability ratios among younger adolescents was limited because prevalence in females was lower than anticipated and true M:F ratios were likely smaller, reflecting sex patterns in young children before adolescence and adulthood patterns emerge. Precision was also reduced among older participants due to unequal recruitment by sex. However, estimates for young adults were relatively more precisely estimated, where both prevalence and M:F ratios were higher.

Second, selection bias cannot be excluded among older adults where men were under-represented. Working-age men are likely to be more mobile and have prevalent infectious TB, but are often under-represented in community-based TB surveys [2, 49]. If indeed older men not enrolled in our survey had high Mtb exposure risks, then our M:F ratios at older ages are underestimates. Sex comparisons were therefore uncertain and should be interpreted with caution. More generally, non-respondents, including those who declined consent or were not included in our sampling frame, could have differed systematically from those enrolled, which cannot be ruled out in the absence of further data. Age-cohort effects and survival bias may also have influenced the observed trajectories, for example, due to interactions between TB and HIV epidemics [54]. Nevertheless, random household sampling produced age and sex distributions broadly consistent with those observed in previous population-based surveys from Malawi [12], supporting the representativeness of our findings. Our selection of 33 high TB burden neighbourhoods of Blantyre City means that findings may not be directly transferable to low burden areas, where absolute prevalence estimates will likely vary; however, we would expect conclusions about age- and sex-specific exposure risks to be relevant for neighbourhoods with similar age- and sex-specific contact patterns.

Third, information bias is possible. Self-reported HIV status, alcohol drinking, and tobacco smoking frequencies may have suffered social desirability bias, potentially differing by sex. Nonetheless, our HIV prevalence estimates align with the 2019–2020 national HIV prevalence survey, which showed a high prevalence of in women among 15–44 year olds; [55] the lower prevalence of HIV among men 20–29 year old compared to 10–19 year olds could be due to underreporting or lack of engagement with HIV testing services in this subgroup. Notably, such underreporting rendered estimates for the HIV association from adjusted models imprecise; a higher than reported HIV prevalence among males implies associations may have been overestimated. Importantly, although the posterior probability of excess male risk decreased at older ages in sensitivity analyses restricted to HIV-negative participants, estimates were largely unchanged for adolescents, suggesting that HIV did not materially influence the observed sex pattern in this age group. In both the 2016 and 2024 national demographic and health surveys, the prevalence of both tobacco smoking and alcohol drinking is higher among women, particularly in urban areas [56, 57], as observed in our study. Any underreporting among women in our study would mean that the associations were overestimated.

Possible residual confounding, due to coarsely measured exposures (e.g. *any lifetime history* of tobacco smoking or alcohol use, household and community-level exposures, etc.) or unmeasured exposures related to contact patterns with individuals with infectious TB, implies that associations observed in this study deviated from true associations: if non-differential, then associations were attenuated, or exaggerated or even reversed if otherwise. Moreover, although desirable, collecting accurate mobility-related exposure data, including public transport use, school and work type, was deemed impractical and at high risk of misclassification, given time and logistical constraints. We were therefore unable to quantify the contributions of these variables to observed sex differences in Mtb immunoreactivity. Needless to say, given the cross-sectional design, associations should not be interpreted as causal.

Finally, our analyses assumed that QFT-Plus positivity reflects prior Mtb exposure. While sex differences in T-cell responses to Mtb antigens could contribute to the observed M:F ratios, independent of true sensitisation status, available evidence indicates that men generally mount weaker interferon gamma responses [58]. Given the high specificity of the QFT-Plus assay and the absence of data suggesting sex-related variation in its performance, differential test reactivity is unlikely to fully explain our findings [59]. The positive associations of QFT-Plus positivity with prior TB treatment and household TB contact, as previously reported, likely represent true Mtb infection, further supporting its validity as a marker of true Mtb infection [60–63]. Previous QFT-Plus surveys have also reported associations with tobacco smoking and alcohol drinking. Moreover, QFT-Plus positivity is not known to be influenced by Bacille Calmette-Guérin vaccination or environmental mycobacterial sensitisation [59]. Finally, the proportion of indeterminate results observed in our study (2.1%) is comparable to those reported in similar studies. For example, among adolescents and young people 15–24 years old in Zambian and South African communities, 2.6% (119/4648) had an indeterminate QFT-Plus result [60]. 

Our M:F ratios of predicted Mtb immunoreactivity probability were not adjusted for reversion because granular age- and sex-specific reversion data are limited. Reversion, the loss of positivity status over time among previously immunoreactive individuals, leads to underestimation of both prevalence and ARTI, particularly among adolescents and adults [34, 35]. We examined the potential impact of IGRA reversion on estimated ARTIs. However, reversion probabilities likely vary by age, sex, and other host characteristics [36], meaning that our reversion-adjusted ARTI estimates may differ from the true values if Malawi-specific reversion rates differ substantially from the South African cohort. Further research is needed to characterise how reversion varies across demographic groups, and to guide how future surveys should incorporate this heterogeneity when modelling Mtb infection dynamics. Similarly, our model-derived age- and sex-specific annual risk of Mtb immunoreactivity conversion estimates inferred from a single survey are prone to cohort and period effects, and do not account for reversion due to a lack of age- and sex-specific reversion data.

Taken together, our findings have implications for TB care and prevention policy and future research. We identified adolescence as a period of increasing Mtb infection risk, when behavioural, biological, and community-level factors converge to increase exposure, particularly among boys and young men. Notably, by allowing for an interaction between age and sex, we showed that the Mtb exposure increases with age, but differently by sex, in contrast to assuming a constant effect of sex across age, as assumed in previous studies [2, 41, 60, 61]. Future Mtb interventions aimed at preventing infection or progression to disease, such as novel vaccines and preventive treatment, may need targeting to ages when exposure risk is highest, possibly by sex. Because recent transmission remains the predominant driver of incidence in high TB burden settings, accurate measurement of transmission and recent infection, beyond Mtb immunoreactivity alone, is essential to quantify Mtb infected individuals at high risk of disease in a given population [64]. Remote infection, which confers increased TB risk even in high incidence settings [65], also warrants further attention. Integrating age- and sex-disaggregated surveillance of Mtb immunoreactivity among adolescents and adults into national TB programmes could help identify priority populations for targeted Mtb infection interventions. This survey informed planning and recruitment for an ongoing adolescent and adult TB vaccine phase 3 clinical trial and should be a useful resource for future studies.

## Conclusions

Our findings suggest that focusing only on reducing sex differences in progression and care access may be too late to interrupt transmission, which is critical for TB elimination. Future research is needed to understand age- and sex-specific vulnerabilities in Mtb exposure to inform global and national TB care and prevention strategies.

## Supplementary Information

Below is the link to the electronic supplementary material.

Supplementary Material 1: Section A–H, Figures S1–S5, Table S1.

## Data Availability

R code and data underlying the findings reported in this manuscript can be found on GitHub (https://github.com/mphadsphiri/MtbBTsex) and Zenodo (DOI: 10.5281/zenodo.20111003) [66].

## Abbreviations

IGRA: Interferon gamma release assay
Mtb: Mycobacterium tuberculosis
QFT-Plus: QuantiFERON-TB Gold Plus
TST: Tuberculin Skin Test
TPT: Tuberculosis preventive therapy
