# Supplementary material for: Drivers of sex differences in Mycobacterium tuberculosis immunoreactivity among adolescents and adults in Blantyre, Malawi
Source: BMC Glob Public Health. 2026 Jul 23;4:68. doi: 10.1186/s44263-026-00302-w (PMC13393826; doi:10.1186/s44263-026-00302-w)
Supplement: Supplementary file 1 — Supplementary Material 1: Section A–H, Figures S1–S5, Table S1. [file 44263_2026_302_MOESM1_ESM.pdf]

Drivers of sex differences in *Mycobacterium tuberculosis* immunoreactivity among adolescents and adults in Blantyre, Malawi

Authors

Mphatso D. Phiri[1,2,3,4] *, Hannah M. Rickman[1,5], Hannah Mbale[1], Helena R.A. Feasey[6], Marriott Nliwasa[7,8], Alvaro Schwalb[9,10,11], Henry C Mwandumba[1,2,3], Elizabeth L Corbett[5], S Bertel Squire[2,3], Marc YR Henrion[1,2,3], Katherine C Horton[9,10], Peter MacPherson[13]

Author affiliations

1. Malawi Liverpool Wellcome Research Programme, Blantyre, Malawi
2. Department of Clinical Sciences, Liverpool School of Tropical Medicine, Liverpool, United Kingdom
3. Centre for TB Research, Liverpool School of Tropical Medicine, Liverpool, United Kingdom
4. School of Medicine and Oral Health, Kamuzu University of Health Sciences, Blantyre, Malawi
5. Clinical Research Department, London School of Hygiene & Tropical Medicine, London, United Kingdom
6. School of Medicine, University of St Andrews, Glasgow, Scotland
7. Helse Nord Clinical Research and Training Initiative, Kamuzu University of Health Sciences, Blantyre, Malawi
8. Division of Epidemiology and Biostatistics, School of Public Health, Faculty of Health Sciences, University of the Witwatersrand, Johannesburg, South Africa
9. TB Modelling Group, TB Centre, London School of Hygiene & Tropical Medicine, London, United Kingdom
10. Department of Infectious Disease Epidemiology, London School of Hygiene & Tropical Medicine, London, United Kingdom

11. Instituto de Medicina Tropical Alexander von Humboldt, Universidad Peruana Cayetano Heredia, Lima, Peru
12. National Tuberculosis and Leprosy Elimination Program, Ministry of Health, Lilongwe, Malawi
13. School of Health and Wellbeing, University of Glasgow, Glasgow, Scotland

* Correspondence: Mphatso D Phiri (mdphiri@m.mw)

Supporting information includes:

1. Section A: Conceptual framework of individual-, household- and community-level risk factors *for Mycobacterium tuberculosis* (Mtb) exposure and infection
2. Section B: Model specification
3. Section C: Participant recruitment
4. Section D: Table S1: Predicted Mycobacterium tuberculosis (Mtb) immunoreactivity probabilities, by age and sex, male-to-female (M:F) ratios comparing predicted probabilities between males and females, and the posterior probability that M:F ratios exceeded 1 at each age.
5. Section E: Mycobacterium tuberculosis immunoreactivity by age and sex according to alcohol drinking and tobacco smoking history predicted from Bayesian logistic regression models
6. Section F: Posterior probability of excess male risk in Mycobacterium tuberculosis immunoreactivity, comparing male-to-female (M:F) predicted probability ratios
7. Section G: Posterior sensitivity to weaker and stronger priors and likelihood
8. Section H: Model comparison, goodness of fit, regression coefficients and for models reported in the main text.

Section A: Conceptual framework of individual-, household- and community-level risk factors for Mycobacterium tuberculosis (Mtb) exposure and infection

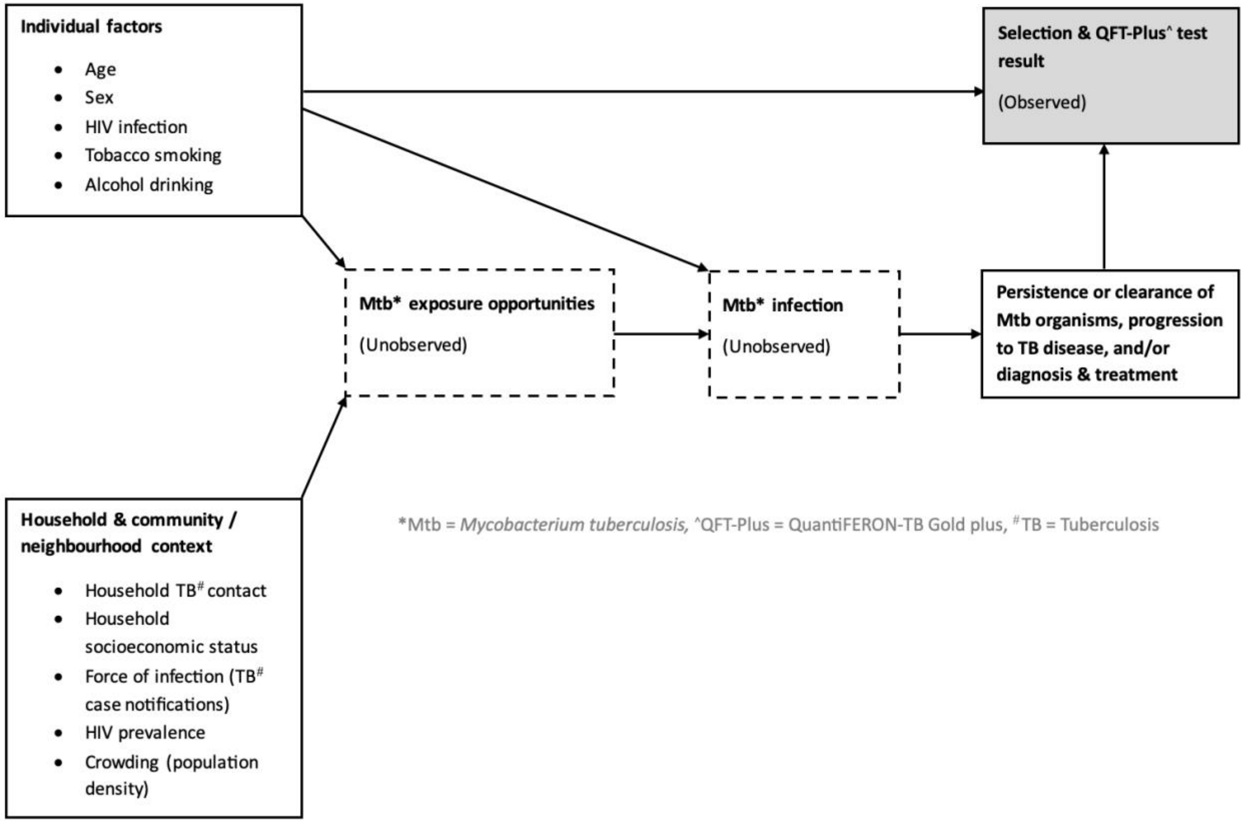

Fig S1: Conceptual framework of risk factors for *Mycobacterium tuberculosis* exposure and infection.

Section B: Model specification

1. “Base” model

Outcome: Mycobacterium tuberculosis (Mtb) immunoreactivity, defined as a positive QuantiFERON-TB Gold Plus test result. Mtb immunoreactivity probability $\Pr(Y_i = 1) = p_i$ for participant $i = 1, \dots, n$ nested in neighbourhood $j = i(j)$ was modelled as a Bernoulli outcome. The likelihood and link were:

$$Y_i \sim \text{Bernoulli}(p_i), \qquad \text{logit}(p_i) = \eta_i.$$

The linear predictor comprises a sex-specific smooth of age and a neighbourhood-level random intercept.

$$\eta_i = \alpha + f_{\text{age}\times\text{sex}}(\text{age}_i, \text{sex}_i) + b_{j[i]}, \qquad b_j \sim \mathcal{N}(0, \sigma_b^2),\ j = 1, \dots, J.$$

The smooth function term for age and sex is specific as a spline basis of dimension K, allowing different age curves by sex:

$$f_{\text{age}\times\text{sex}}(\text{age}, \text{sex}) = \sum_{k=1}^{K} \gamma_k\ B_k(\text{age}) + \mathbb{1}\{\text{sex} = \text{male}\} \sum_{k=1}^{K} \delta_k\ B_k(\text{age}),$$

where $B_k(\cdot)$ are basis functions (thin-plate regression splines in implementation), γ_k the baseline smooth coefficients and δ_k the male-specific deviations. K values were compared via leave-one-out cross-validation to identify an adequate spline basis dimension.

2. “Full” model (adding individual & neighbourhood-level covariates)

We let $\mathbf{x}_i$ represent individual-level covariates (HIV status, previous TB treatment, household TB contact, smoking, alcohol); membership in the poorest poverty quartile of households; all specified as binary variables, and neighbourhood -level covariates (2020 TB case notification rate (mean-centred), 2019 adult HIV prevalence, population density (mean-centred)), with corresponding coefficients $\boldsymbol{\beta}$. The full model extended the linear predictor as:

$$\eta_i = \alpha + f_{\text{age}\times\text{sex}}(\text{age}_i, \text{sex}_i) + \mathbf{x}_i^\top \boldsymbol{\beta} + b_{j[i]}.$$

3. Priors

Priors were weakly informative, specified as below, visualised via prior predictive checks to inspect their implied probabilities, and prior–data conflict and sensitivity were examined using the **priorsense** package v1.1.0:

- Intercept (logit scale) centred on a baseline prevalence of 20% in females:

$$\alpha \sim \mathcal{N}(\text{logit}(0.2),\, 1^2) \quad \text{with} \quad \text{logit}(0.2) = \log\frac{0.2}{0.8} \approx -1.386$$

- Population-level coefficients:

$$\beta_m \sim \mathcal{N}(0, 1^2) \text{ for each } m,$$

- Neighbourhood-level random-intercept standard deviation:

$$\sigma_b \sim \text{Exponential}(1).$$

- Smooth (spline) standard deviations (brms class = sds, lower bounded at 0):

$$\sigma_f \sim \text{Half-Student-}t(\nu = 2,\, 0,\, 2).$$

where σ_b is the SD of the neighbourhood random intercepts, and σ_f is the SD parameter governing the spline penalties. In brms, priors on SD parameters are constrained to be non-negative; thus the Student-t prior on σ_f is implicitly half-t.

4. Estimation

Models were fitted in a Bayesian framework using Hamiltonian Monte Carlo via the brms (v2.22.0) interface to Stan in the R environment for statistical computing (v4.3.3). We run 4 chains with 2,000 iterations per chain (1,000 warm-up), yielding 4,000 post-warm-up draws. Convergence and mixing were assessed using $\widehat{\text{R}}$, effective sample sizes, and visual inspection of

trace plots. We set a conservative target acceptance probability (e.g., adapt_delta = 0.999) to avoid divergent transitions. Models were fitted in R v4.3.3 using the brms package v2.22.0, an interface to Stan v0.7.1 implementing Hamiltonian Monte Carlo.

5. Posterior summaries and predictions

Posterior estimates are reported as posterior means and 95% credible intervals (CrIs; central 95% posterior percentiles). For age- and sex-specific inferences we calculated predictions, excluding neighbourhood-level random effects by setting $b_j = 0$ (population-level predictions).

6. Annual risk of Mtb immunoreactivity conversion (ARC)

Let $p(a, s)$ denote the predicted probability of immunoreactivity at age a and sex s. The **annual risk of conversion (ARC)** at age a was defined as:

$$\mathrm{ARC}(a, s) \ = \frac{p(a+1, s) - p(a, s)}{1 - p(a, s)} \times 100\%.$$

To quantify sex differences we computed **male-to-female (M:F) ratios** of predicted probabilities and ARCs at each age:

$$\mathrm{M{:}F}_p(a) \ = \frac{p(a,\text{male})}{p(a,\text{female})}, \qquad \mathrm{M{:}F}_{\mathrm{ARC}}(a) \ = \frac{\mathrm{ARC}(a,\text{male})}{\mathrm{ARC}(a,\text{female})}.$$

The **posterior probability of excess male risk** was reported as $\Pr\{\mathrm{M{:}F}_p(a) > 1 \mid \text{data}\}$, computed as the proportion of posterior draws where the ratio exceeded 1 (with Monte Carlo standard error).

7. Interpretation

We exponentiated logit scale fixed-effect coefficients to obtain odds ratios. Nonlinear age effects are obtained from probabilities obtained via the logit-inverse function to the odds; uncertainty in both the smooth and random effects is propagated through all posterior summaries.

Section C: Participant recruitment

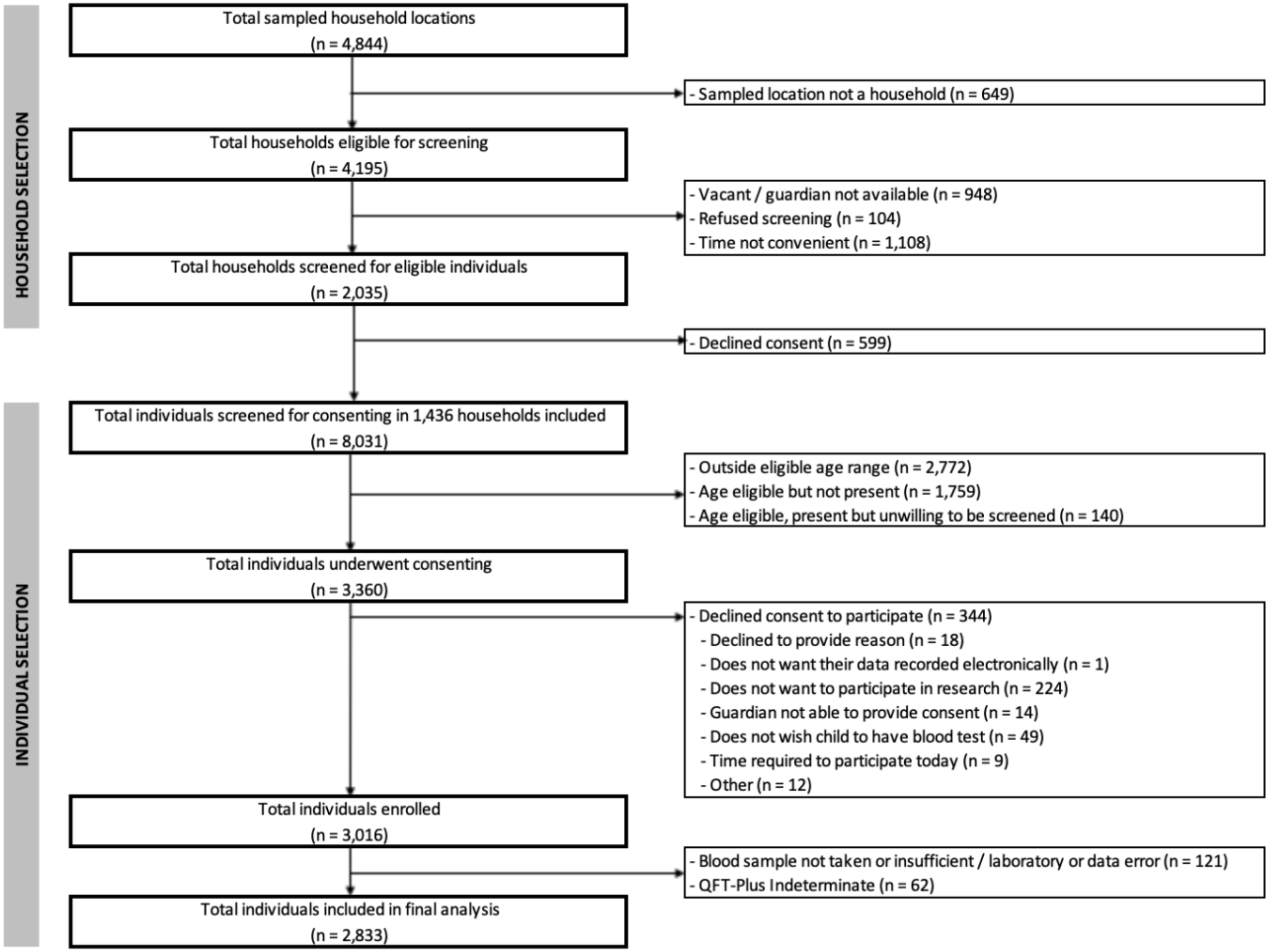

Fig S2: Participant recruitment

Section D: Mycobacterium tuberculosis immunoreactivity probabilities and male-to-female ratios

Table S1: Predicted *Mycobacterium tuberculosis* (Mtb) immunoreactivity probabilities, by age and sex, male-to-female (M:F) ratios comparing predicted probabilities between males and females, and the posterior probability that M:F ratios exceeded 1 at each age.

Age (years)	Predicted probability of Mtb immunoreactivity[1] (%)		M:F[2] probability ratio (95% CrI)	Posterior probability[3] that M:F ratio exceeded 1	MCSE[4] of posterior probability M:F > 1
	Male	Female			
10	6.2% (3.9%-9.1%)	8.5% (5.7%-11.8%)	0.74 (0.44-1.18)	9.9%	0.0052
11	6.9% (4.6%-9.6%)	9.1% (6.4%-12.2%)	0.77 (0.49-1.14)	9.3%	0.0049
12	7.6% (5.4%-10.2%)	9.8% (7.2%-12.6%)	0.79 (0.55-1.11)	8.6%	0.0046
13	8.5% (6.3%-10.9%)	10.5% (8.1%-13.1%)	0.82 (0.60-1.09)	7.8%	0.0044
14	9.4% (7.4%-11.7%)	11.2% (9.0%-13.6%)	0.85 (0.67-1.06)	7.2%	0.0043
15	10.4% (8.5%-12.5%)	11.9% (9.9%-14.2%)	0.88 (0.73-1.05)	7.1%	0.0045
16	11.5% (9.8%-13.5%)	12.7% (10.9%-14.8%)	0.91 (0.79-1.04)	7.8%	0.0050
17	12.7% (11.0%-14.6%)	13.6% (11.8%-15.5%)	0.94 (0.83-1.05)	12.6%	0.0061
18	13.9% (12.2%-15.9%)	14.4% (12.7%-16.3%)	0.97 (0.87-1.08)	26.2%	0.0074
19	15.2% (13.2%-17.4%)	15.3% (13.5%-17.2%)	0.99 (0.88-1.12)	45.8%	0.0083
20	16.5% (14.2%-19.0%)	16.2% (14.3%-18.3%)	1.02 (0.89-1.17)	60.2%	0.0087
21	17.8% (15.1%-20.7%)	17.1% (15.0%-19.4%)	1.05 (0.89-1.22)	68.8%	0.0091
22	19.1% (16.1%-22.4%)	17.9% (15.7%-20.5%)	1.07 (0.89-1.27)	74.4%	0.0091
23	20.4% (17.1%-24.1%)	18.8% (16.4%-21.5%)	1.09 (0.89-1.31)	78.8%	0.0087
24	21.6% (18.0%-25.7%)	19.6% (17.1%-22.6%)	1.11 (0.90-1.34)	81.8%	0.0079
25	22.8% (18.9%-27.1%)	20.4% (17.7%-23.6%)	1.12 (0.90-1.36)	83.9%	0.0072

26	23.9% (19.9%-28.4%)	21.2% (18.4%-24.5%)	1.13 (0.91-1.38)	86.2%	0.0066
27	24.9% (20.8%-29.5%)	22.0% (19.1%-25.2%)	1.14 (0.92-1.38)	87.8%	0.0062
28	25.8% (21.8%-30.4%)	22.7% (19.8%-25.9%)	1.14 (0.93-1.38)	89.9%	0.0053
29	26.6% (22.6%-31.2%)	23.3% (20.4%-26.6%)	1.15 (0.94-1.37)	91.6%	0.0045
30	27.4% (23.3%-31.9%)	23.9% (21.0%-27.2%)	1.15 (0.95-1.37)	92.8%	0.0043
31	28.0% (23.8%-32.6%)	24.5% (21.6%-27.6%)	1.15 (0.96-1.36)	92.8%	0.0045
32	28.6% (24.2%-33.4%)	25.0% (22.1%-28.1%)	1.14 (0.95-1.36)	92.2%	0.0050
33	29.0% (24.3%-34.3%)	25.5% (22.5%-28.8%)	1.14 (0.93-1.37)	90.1%	0.0055
34	29.5% (24.1%-35.3%)	26.0% (22.7%-29.5%)	1.14 (0.91-1.39)	86.8%	0.0061
35	29.8% (23.8%-36.3%)	26.5% (22.8%-30.3%)	1.13 (0.88-1.42)	82.7%	0.0068
36	30.1% (23.3%-37.5%)	26.9% (22.7%-31.2%)	1.13 (0.84-1.45)	78.4%	0.0070
37	30.4% (22.7%-38.9%)	27.3% (22.6%-32.3%)	1.12 (0.80-1.49)	74.0%	0.0073
38	30.7% (21.9%-40.2%)	27.7% (22.4%-33.4%)	1.12 (0.76-1.54)	70.0%	0.0076
39	30.9% (21.1%-41.9%)	28.2% (22.1%-34.6%)	1.11 (0.72-1.59)	66.8%	0.0078
40	31.2% (20.4%-43.4%)	28.6% (21.8%-35.8%)	1.11 (0.68-1.65)	63.6%	0.0078

1. Mtb: *Mycobacterium tuberculosis* (Mtb) immunoreactivity probability predicted from a model adjusted for age and sex, with age specified via a thin-plate regression spline function, separately for each sex. Mtb immunoreactivity was defined as a positive QFT-Plus test.
2. M:F: ratio comparing predicted Mtb immunoreactivity probability between males and females
3. The proportion of M:F ratios that exceeded 1 across 4,000 posterior draws, expressed as a percentage. M:F exceeding 1 denotes excess male risk, and excess female risk below 1.
4. Monte Carlo standard error of the proportion of posterior M:F ratios exceeding 1.

Section E: Mycobacterium tuberculosis immunoreactivity by age and sex according to alcohol drinking and tobacco smoking history predicted from Bayesian logistic regression models

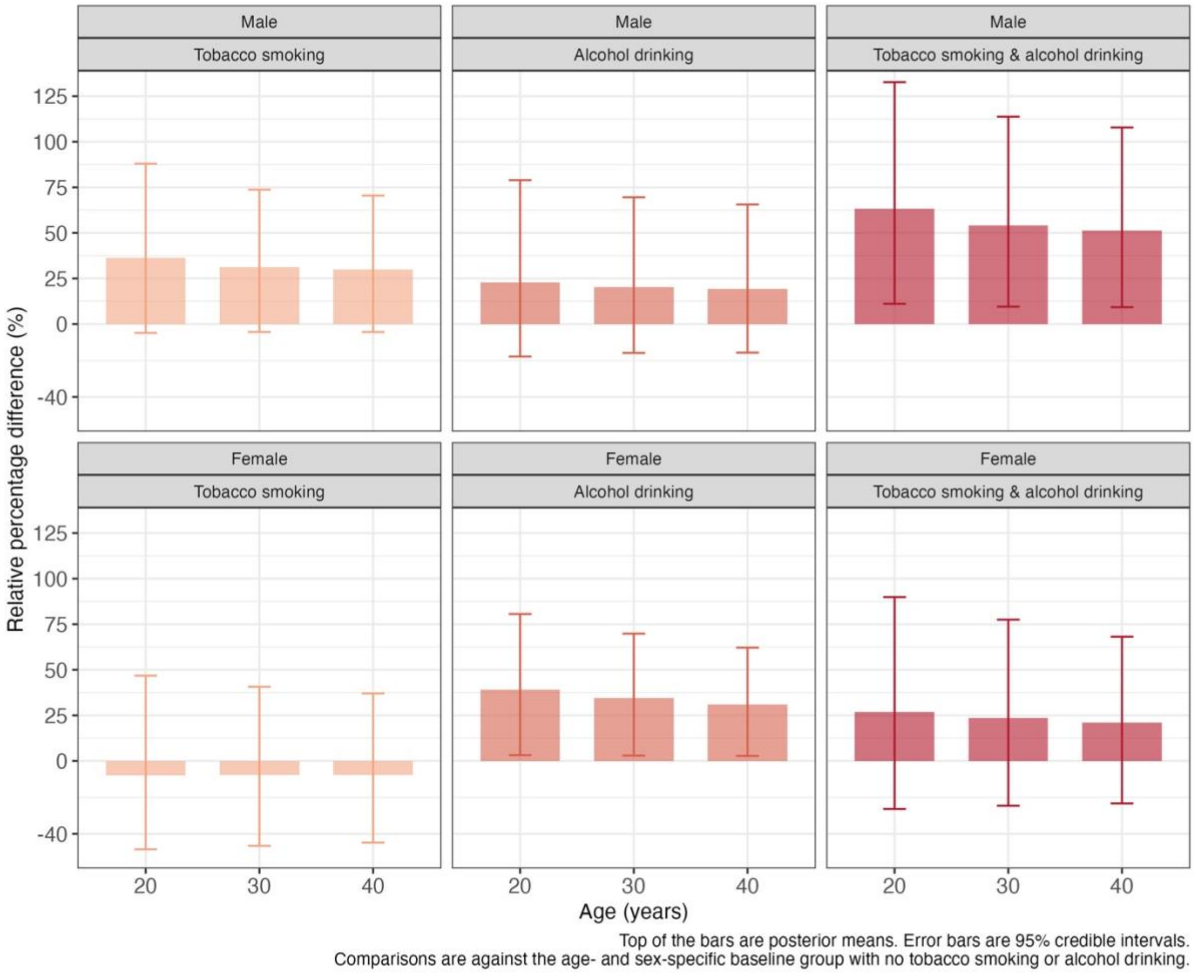

Fig S3: Relative percentage point change in predicted Mycobacterium tuberculosis (Mtb) immunoreactivity probability comparing individuals with any lifetime alcohol drinking or tobacco smoking history at selected ages by sex. The baseline group was HIV negative individuals without previous TB treatment or household TB contact history, without any lifetime history of alcohol drinking or tobacco smoking. Probabilities were exponentiated odds of Mtb immunoreactivity estimated from a Bayesian logistical regression model adjusted for age, sex, HIV status, previous TB treatment history, household TB contact history, any lifetime alcohol drinking and any lifetime tobacco smoking. Age was included as a thin-plate spline separately for each sex. Alcohol drinking and tobacco smoking were included as fixed and interaction terms with sex, respectively. Mtb immunoreactivity was defined as a positive QuantiFERON-TB Gold Plus test. HIV status was self-reported. The relative percentage point change was calculated as the predicted probability in the comparison group minus the baseline group, divided by the baseline group x 100. Top of the bars are means. Error bars are 95% credible intervals.

Section F: Posterior probability of excess male risk in *Mycobacterium tuberculosis* immunoreactivity, comparing male-to-female (M:F) predicted probability ratios

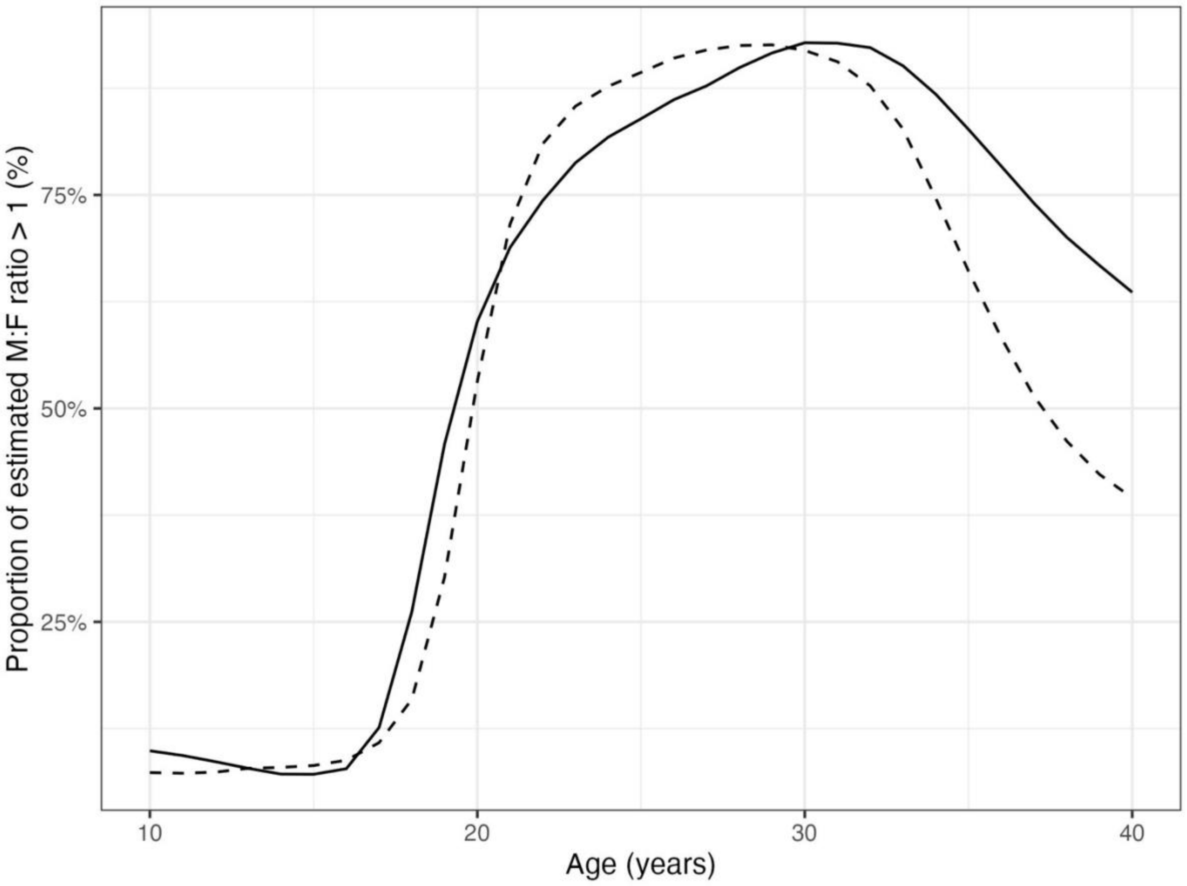

Fig S4: Posterior probability that male-to-female (M:F) ratio of Mtb immunoreactivity probability exceed 1 across age. Probabilities were predicted from a Bayesian logistical regression model of Mtb immunoreactivity additionally adjusted for age, sex and a neighbourhood level random effect, but predictions excluded random effects. M:F ratios were calculated at each posterior draw. Mtb immunoreactivity was defined as a positive QFT-Plus test. Posterior probability is the proportion of M:F ratios exceeding 1 across 4,000 draws. Solid lines are predictions from a model fitted to both HIV negative and HIV positive participants. Dashes lines are predictions from a model fitted to HIV negative participants only.

Section G: Posterior sensitivity to weaker and stronger priors and likelihood

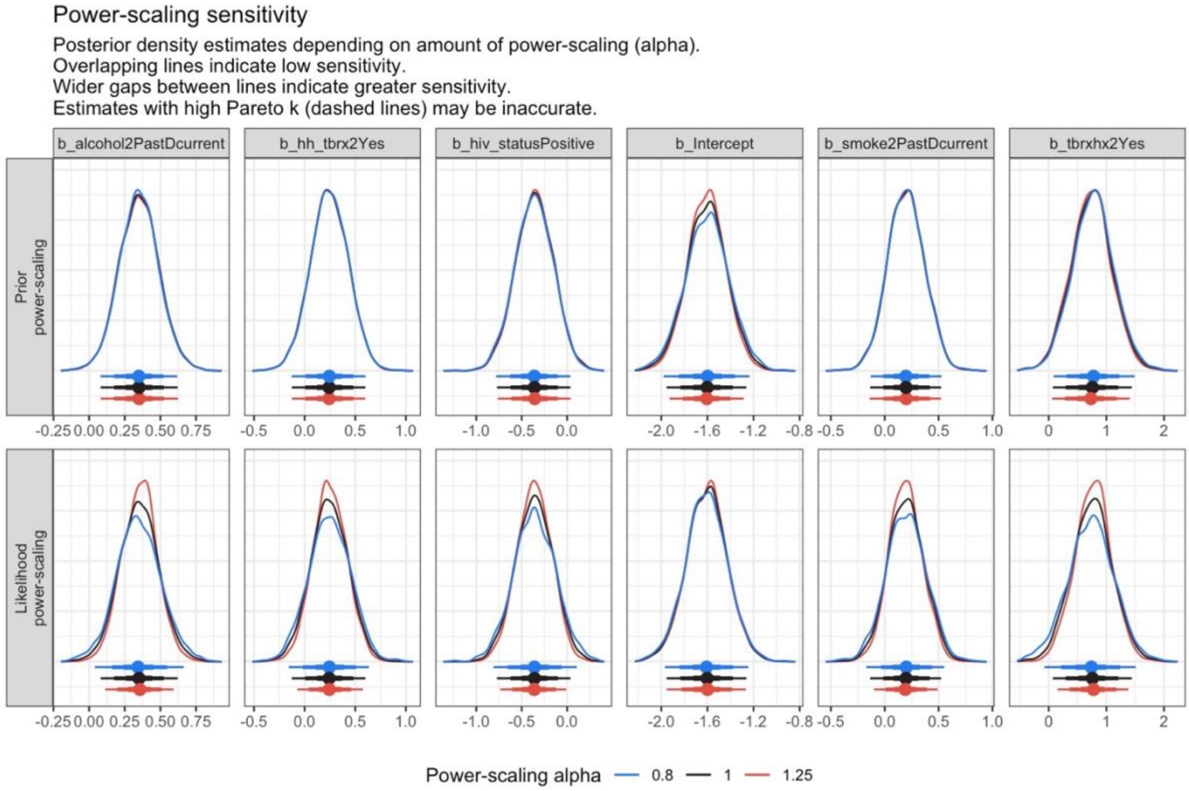

Fig S5: Density plots showing model posterior sensitivity to weaker and stronger priors and likelihood for fixed model effects. Plots are outputs from the priorsense R package.

Section H: Model comparison, goodness of fit, regression coefficients and for models reported in the main text.

1. Models and covariates

Several multilevel Bayesian logistic regression models were fitted to estimate the odds of Mtb immunoreactivity. Covariates included individual variables: age (integer years); sex (male/female); HIV status (positive/negative), previous TB treatment, household TB contact, (as indicator variables: (yes/no); and any lifetime smoking, and any lifetime alcohol drinking (as indicator variables: ("past or current"/never); household variables: membership in the poorest quartile of households (yes/no); and neighbourhood variables: 2019 neighbourhood adult HIV prevalence, 2020 neighbourhood TB case notification rates (mean scaled), and neighbourhood population density (mean scaled). Age was specified via a thin-plate regression spline function, for each sex. All models included a neighbourhood random effect to account for the cluster household sampling.

2. Spline specification for age variable

Models with age specified as a continuous, 5- or 10-year group variable (age_5y or age_10_y), or as an integer via a thin-plate spline function (spline_k3, …, spline_k6), were compared via leave-one-out cross-validation.

model	elpd_diff	se_diff	elpd_loo	se_elpd_loo	p_loo	se_p_loo	looic	se_looic
spline_k3	0.00	0.00	-1277.59	31.2	8.95	0.3	2555.18	62.4
age_10y	-9.38	3.98	-1286.97	31.3	8.8	0.28	2573.94	62.6
age_5y	-6.00	2.27	-1283.58	31.18	11.97	0.38	2567.17	62.36
continuous	-4.75	3.07	-1282.34	31.21	7.74	0.25	2564.68	62.43
spline_k5	-1.49	1.29	-1279.08	31.11	9.47	0.31	2558.16	62.21
spline_k4	-1.43	1.26	-1279.01	31.09	9.07	0.3	2558.03	62.17
spline_k6	-0.79	0.79	-1278.38	31.18	9.92	0.33	2556.76	62.36

3. Covariate selection and model comparison

Models were adjusted for age and sex *a priori*, and included different sets of variables, as follows:

1. m0_age_sex : age (integer years) and sex (male/female).
2. m1_full: m0_age_sex additionally adjusted for HIV status (positive/negative), previous TB treatment, household TB contact, (as indicator variables: (yes/no); and any lifetime smoking, and any lifetime alcohol drinking (as indicator variables: ("past or current"/never)
3. m1_full_popdens: m1_full additionally adjusted for neighbourhood population density
4. m1_full_cluster_pov: m1_full additionally adjusted for membership in the poorest quartile of households.
5. m1_full_cnr: m1_full additional adjusted for neighbourhood TB case notification rates
6. m1_full_cluster_hiv: m1_full additionally adjusted for neighbourhood adult HIV prevalence.
7. m2_full: m1_full additionally adjusted for neighbourhood adult HIV prevalence, neighbourhood TB case notification rates, and neighbourhood population density.

4. Model comparison

Table below shows model comparison statistics for fitted models.

model	looic	looic_se	p_loo	se_p_loo	elpd_diff	se_diff
m1_full_popdens	1960.7	50.5	14.4	0.6	0.0	0.0
m1_full_cnr	1964.9	50.5	15.6	0.6	-2.1	2.1
m2_full	1965.3	50.7	17.6	0.7	-2.3	0.9
m1_full_cluster_hiv	1965.4	50.3	15.3	0.6	-2.3	2.4
m1_full	1966	50.3	15.4	0.6	-2.6	2.4
m1_full_pov	1967.4	50.4	16.3	0.6	-3.3	2.5

Full model regression coefficients (log scale) are presented below. For each model, mean, sd, q2.5 and q97.54 are presented up to 4 decimal points, and ess_bulk and ess_tail are rounded to whole number.

model	variable	mean	sd	q2.5	q97.5	rhat	ess_bulk	ess_tail
m0_age_sex	b_Intercept	-1.6041	0.0579	-1.7196	-1.4901	1.0011	3905	2797
m0_age_sex	bs_sage:sexFemale_1	0.3990	0.0630	0.2749	0.5226	0.9995	4485	3203
m0_age_sex	bs_sage:sexMale_1	0.5279	0.0866	0.3565	0.6995	1.0014	5303	2646
m0_age_sex	sd_cluster__Intercept	0.1032	0.0703	0.0045	0.2532	1.0052	1288	1923
m0_age_sex	sds_sagesexFemale_1	2.1744	1.9882	0.1309	7.1894	1.0011	2020	1713
m0_age_sex	sds_sagesexMale_1	3.0920	2.6811	0.4043	9.8916	1.0004	2015	1533
m0_age_sex	Intercept	-1.6041	0.0579	-1.7196	-1.4901	1.0011	3905	2797
m0_age_sex	s_sagesexFemale_1[1]	1.2993	0.9586	-0.2292	3.2481	1.0023	2585	2131
m0_age_sex	s_sagesexMale_1[1]	2.4370	1.3038	0.0295	4.9491	1.0005	2570	1432
m1_full	b_Intercept	-1.6050	0.0846	-1.7744	-1.4436	1.0006	4745	3078
m1_full	b_hiv_statusPositive	-0.3604	0.2091	-0.7787	0.0404	1.0007	5626	3088
m1_full	b_smoke2PastDcurrent	0.2148	0.1744	-0.1324	0.5409	1.0029	5848	3105
m1_full	b_alcohol2PastDcurrent	0.3381	0.1361	0.0775	0.6036	1.0019	4691	3109
m1_full	b_tbrxhx2Yes	0.7749	0.3579	0.0824	1.4710	1.0010	6615	3440
m1_full	b_hh_tbrx2Yes	0.2572	0.1851	-0.1151	0.6126	1.0006	5810	3383

m1_full	bs_sage:sexFemale_1	0.3899	0.0828	0.2296	0.5582	1.0017	5283	3185
m1_full	bs_sage:sexMale_1	0.4168	0.1070	0.2054	0.6267	1.0014	5877	2800
m1_full	sd_cluster__Intercept	0.1326	0.0868	0.0056	0.3235	1.0013	1315	2015
m1_full	sds_sagesexFemale_1	1.8971	1.9514	0.0757	6.9557	1.0008	2492	2065
m1_full	sds_sagesexMale_1	2.6811	2.3896	0.1552	9.4623	1.0030	2020	1294
m1_full	Intercept	-1.4515	0.0682	-1.5865	-1.3189	1.0002	4365	2783
m1_full	s_sagesexFemale_1[1]	0.9474	0.9605	-0.498	3.1063	1.0001	2862	2991
m1_full	s_sagesexMale_1[1]	1.9540	1.4361	-0.3232	4.9086	1.0031	2474	2457
m1_full_popdens	b_Intercept	-1.6127	0.0820	-1.7760	-1.4538	1.0020	4458	3244
m1_full_popdens	b_hiv_statusPositive	-0.3433	0.2116	-0.7679	0.0562	1.0006	5189	2954
m1_full_popdens	b_smoke2PastDcurrent	0.2068	0.1727	-0.1407	0.5419	1.0007	3885	2875
m1_full_popdens	b_alcohol2PastDcurrent	0.3461	0.1390	0.0689	0.6129	1.0008	3730	2879
m1_full_popdens	b_tbrxhx2Yes	0.7455	0.3658	0.0299	1.4771	1.0006	4071	2838
m1_full_popdens	b_hh_tbrx2Yes	0.2492	0.1877	-0.1310	0.6116	1.0000	4163	2955
m1_full_popdens	b_pop_dens_km2_std	0.1626	0.0611	0.0448	0.2849	1.0002	4481	3210
m1_full_popdens	bs_sage:sexFemale_1	0.3932	0.0852	0.2276	0.5676	1.0015	3801	3061

m1_full_popdens	bs_sage:sexMale_1	0.4253	0.1071	0.2249	0.6420	1.0008	4211	2579
m1_full_popdens	sd_cluster__Intercept	0.1035	0.0726	0.0049	0.2699	1.0027	1408	1924
m1_full_popdens	sds_sagesexFemale_1	1.9643	2.0563	0.0622	6.9846	1.0031	1737	1210
m1_full_popdens	sds_sagesexMale_1	2.7011	2.5854	0.1195	9.1112	1.0002	1681	1077
m1_full_popdens	Intercept	-1.4591	0.0638	-1.5846	-1.3336	1.0015	3979	3255
m1_full_popdens	s_sagesexFemale_1[1]	1.0144	0.9732	-0.5286	3.1745	1.0016	2501	3367
m1_full_popdens	s_sagesexMale_1[1]	1.8888	1.3845	-0.2665	4.7994	1.0009	1868	2684
m2_full	b_Intercept	-1.6051	0.1760	-1.9543	-1.2619	1.0019	5594	3009
m2_full	b_hiv_statusPositive	-0.3605	0.2062	-0.7733	0.0363	1.0019	5891	3127
m2_full	b_smoke2PastDcurrent	0.1984	0.1686	-0.1353	0.5234	1.0008	3775	3314
m2_full	b_alcohol2PastDcurrent	0.3509	0.1380	0.0800	0.6217	1.0005	3736	2880
m2_full	b_tbrxhx2Yes	0.7524	0.3537	0.0699	1.4414	1.0019	5205	3202
m2_full	b_hh_tbrx2Yes	0.2447	0.1843	-0.1271	0.6008	1.0015	4804	3229
m2_full	b_poorest_quartileYes	0.0625	0.1270	-0.1790	0.3136	1.0008	5659	3262
m2_full	b_cnr_std	0.0237	0.0699	-0.1092	0.1592	1.0005	3565	3239
m2_full	b_cluster_hiv_prev	-0.1692	0.9549	-2.0243	1.7531	1.0035	5981	2921

m2_full	b_pop_dens_km2_std	0.1492	0.0728	0.0089	0.2885	1.0008	3244	3083
m2_full	bs_sage:sexFemale_1	0.4005	0.0822	0.2413	0.5603	1.0004	4170	3062
m2_full	bs_sage:sexMale_1	0.4270	0.1059	0.2203	0.6338	1.0017	5033	3268
m2_full	sd_cluster__Intercept	0.1071	0.0762	0.0040	0.2846	1.0025	1433	1694
m2_full	sds_sagesexFemale_1	1.9847	2.1157	0.0734	6.8834	1.0010	1843	2082
m2_full	sds_sagesexMale_1	2.6714	2.6620	0.1556	9.4086	1.0001	1866	1329
m2_full	Intercept	-1.4624	0.0654	-1.5902	-1.3317	1.0010	4266	2959
m2_full	s_sagesexFemale_1[1]	1.0090	0.9746	-0.5042	3.1408	1.0000	2308	3470
m2_full	s_sagesexMale_1[1]	1.8858	1.4114	-0.3362	4.8188	1.0002	2015	2130
